# Single crystal structure, vibrational spectroscopy, gas sorption and antimicrobial properties of a new inorganic acidic diphosphates material (NH_4_)_2_Mg(H_2_P_2_O_7_)_2_•2H_2_O

**DOI:** 10.1038/s41598-020-65718-2

**Published:** 2020-06-01

**Authors:** Rachid Essehli, Souhir Sabri, Fedwa El-Mellouhi, Brahim Aïssa, Hamdi Ben Yahia, Tausif Altamash, Majeda Khraisheh, Abdulkarem Amhamed, Brahim El Bali

**Affiliations:** 1 Energy and Transportation Science Division, Oak Ridge National Laboratory, Oak Ridge, TN USA; 2 Qatar Environment & Energy Research institute (QEERI), Hamad Bin Khalifa University (HBKU), Qatar Foundation, P.O. Box 34110 Doha, Qatar; 3 Chemical Engineering Department, Qatar University, P.O. Box 2713 Doha, Qatar

**Keywords:** Materials science, Materials for energy and catalysis, Nanoscale materials, Structural materials, Theory and computation

## Abstract

We report on the successful synthesis of diammonium magnesium dihydrogendiphosphate (V) dihydrate compound (NH_4_)_2_Mg(H_2_P_2_O_7_)_2_•2H_2_O using a wet chemical route. Single crystal X-ray diffraction analysis and micro Raman spectroscopy are employed to characterize the compound. We demonstrate, using a multidisciplinary approach, that this compound is ideal for carbon dioxide (CO_2_) capture in addition to other anthropogenic gasses. We show here -from both an experimental as well as from a density functional theory (DFT) calculations routes- the potential for adopting this compound into domestic air-conditioning units (ACUs). From these experiments, the resistance to bacterial growth is also investigated, which is critical for the adoption of this compound in ACUs. Our  compound exhibits a higher methane (CH_4_) sorptivity as compared to CO_2_ at 25 °C and 45 °C under pressures up to 50 bars. Furthermore, DFT electronic structure calculations are used to compute the main structural and electronic properties of the compound, taking into consideration the characteristics of the identified pores as a function of the progressive CO_2_ vs. CH_4_ loadings. Finally, the antibacterial assay reveals a strong antibacterial activity against the tested Gram-positive and Gram-negative bacteria, with a large zone of inhibition against the tested *E*. *Coli*, *S. Aureus* and *K. Pneumonia*.

## Introduction

The (NH_4_)_2_Mg(H_2_P_2_O_7_)_2_•2H_2_O compound belongs to the family of the inorganic acidic diphosphates with general formula *A*_2_*M*(H_2_P_2_O_7_)_2_•nH_2_O, where *A* is an ammonium or an alkaline cation, and *M* a divalent 3d cation, in this case Mg^2+^, (further abbreviated as *MTPy*•nH_2_O). These compounds hold important biochemical roles, such as being an inhibitor of human immunodeficiency enzymes^1^ and of the formation and dissolution of apatite crystals^2^. Furthermore, they are used in piezoelectric, luminescent, ceramic and solid-state laser applications^3–9^. Recently, our research team reported on the structure of *A*_2_*M*(H_2_P_2_O_7_)_2_•nH_2_O compounds, where *A* is an ammonium or an alkaline cation, and *M* a divalent 3d cation or Zn^2+^ and Mg^2+^. These materials are polymorphic structures with three different space groups, namely P-1, Pnma and P2/m (see Table S1)^10–17^. Many inorganic acidic diphosphates compounds containing H_2_P_2_O_7_, HP_2_O_7_ and P_2_O_7_ fragments exhibiting important biological properties such as antitumor, antibacterial and antifungal activity against *Salmonella typhimurium*, *Enterococcus feacium* and *Candida albicans*^18^.

Due to human activities such as the burning of fossil fuels and biomass, deforestation, rice cultivation, domestic ruminant rearing, coal mining and natural gas venting, the CO_2_ and CH_4_ concentrations in the atmosphere are increasing significantly leading to increased global warming causing climate change. This represents a global threat to humanity with CO_2_ and CH_4_ identified as the main man-made anthropogenic greenhouse gases^19^. However, due to the depletion of petroleum, natural - and biogas are becoming more important energy resources. The causal effect is a surge in new technologies developed around the use of gases, fuels and feedstock chemicals^20^. The removal of any individual gas from flue gas, biogas, or landfill gases, through gas absorption techniques, is a crucial step^21–24^, and improving the CO_2_ and CH_4_ capture efficiency is a priority. Indeed, the separation/storage processes for CO_2_ and CH_4_ gases require the establishment of efficient strategies to reduce energy consumption and cost, through innovative absorbents and technologies. For this aim, various types of materials have been investigated so far for sorption purposes, including activated carbon^25^, carbon molecular sieves^26^, zeolites, metal organic frameworks (MOFs)^21,27^ and room temperature liquid materials (ionic liquids and deep eutectic solvents)^28–32^. Unfortunately, until recently, the deployment of an efficient technology based on the physi-sorbents such as (MOFs), and zeolites are hindered by poor selectivity for CO_2_ over the other components of industrial gas mixtures.

In this work, we report the synthesis of a (NH_4_)_2_Mg(H_2_P_2_O_7_)_2_•2H_2_O compound via a wet chemical route and its capacity for CO_2_ and CH_4_ sorption. The crystal structure was determined using the single X-ray diffraction with Raman and IR analyses used to point out the internal and external vibrational modes for (H_2_P_2_O_7_)^2-^, (NH_4_)^+^ and H_2_O and Mg^2+^ in the unit cell of the crystal (NH_4_)_2_Mg (H_2_P_2_O_7_)_2_•2H_2_O.

Furthermore, the CO_2_/CH_4_ adsorption capability of (NH_4_)_2_Mg(H_2_P_2_O_7_)_2_•2H_2_O was explored together with its potential use as a bacterial inhibition zone against *E. Coli, S. Aureus and K. Pneumonia* while assessing its safe incorporation into domestic air-conditioning units enabling to mitigate bacterial growth and proliferation^18^. To complement our experimental investigation, we employed DFT calculations to study the structural and electronic properties of the material such as the band structure and the projected electronic density of states (PDOS). In addition, we explored the landscape of pores in (NH_4_)_2_Mg(H_2_P_2_O_7_)_2_ identifying those with a pore radius> 1 Å and their energetics upon progressive CO_2_ vs. CH_4_ loading. This compound showed a clear potential for CO_2_/CH_4_ adsorption and storage thus opening the way for its exploration within air conditioning systems, through its adaptation for capturing and collecting carbon dioxide and greenhouse gases from the air, and their conversion into hydrocarbon fuels using existing mature technologies.

## Results and Discussion

### X-ray crystal structure

**Figure 1:**
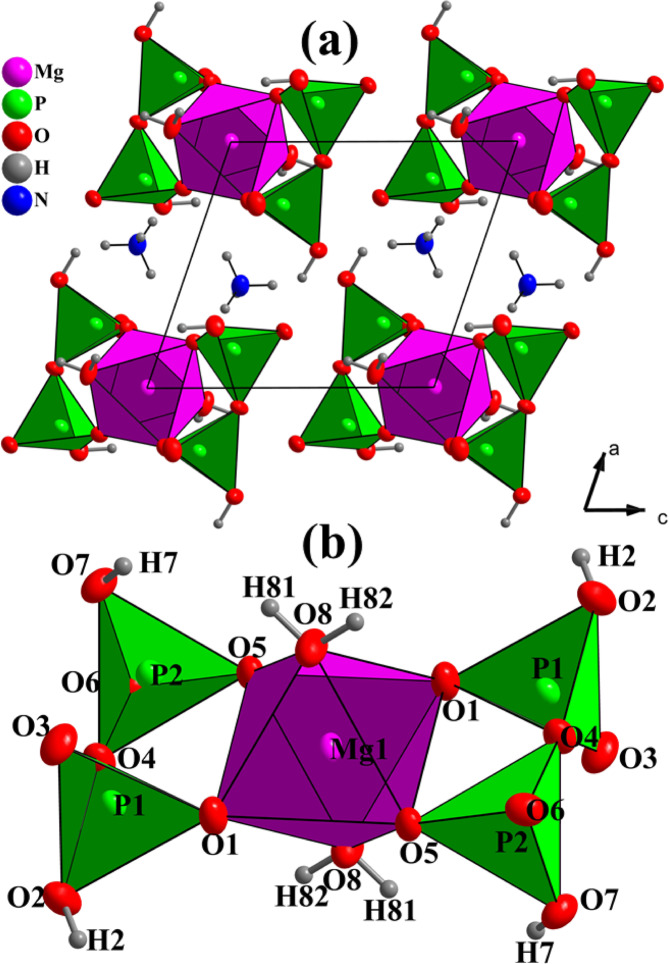
(**a**) Projection of the crystal structure along [010], and (**b**) view of the Mg(H_2_P_2_O_7_)_2_•2H_2_O unit. The structural graphics were created with DIAMOND program.

(NH_4_)_2_Mg(H_2_P_2_O_7_)_2_•2H_2_O is isostructural with its zinc homologue^14^, the crystal structure is mainly built of isolated [Mg(H_2_P_2_O_7_)_2_•2H_2_O]^2-^ anions separated by NH_4_^+^ cations (the schematic is displayed in Fig. 1a,b). The Mg atom lies on the inversion center (0, 1/2, 0) and is octahedraly coordinated by four O atoms from two bidendate H_2_P_2_O_7_ groups, and two water molecules in trans positions with respect to the basal plane containing phosphorous and magnesium atoms (Fig. 1b). Selected interatomic distances, angles and bond valences of (NH_4_)_2_Mg(H_2_P_2_O_7_)_2_•2H_2_O as measured by XRD and calculated by DFT are shown in Tables S2–S5 (average distances and angles are given under brackets.)

Within the Mg octahedron, Mg‒O_p_ (phosphate) bond distances of 2.077(3) and 2.086(3) Å are shorter than the Mg‒O_w_ (water) distance of 2.110(3) Å, which is in agreement with the lower Lewis basicity of the water oxygens with respect to those of oxoanions^8^. [MgO_6_] is regular, with O8 water oxygens in apical positions, resembling a trans- [MgO_4_(H_2_O)_2_] octahedron (D4h idealized symmetry). The distortion index, formulated by [(Mg–O)_max_–(Mg‒O) _min_]/<Mg‒O > , where the mean average Mg‒O distance is 0.015^8^, which is typical of Mg octahedra. The low value is basically due to the short Mg^2+^ ionic radius (0.72 Å). The MgO_6_ octahedra are isolated in the structure, with a Mg‒Mg distance exceeding 7.067 Å.

The (H_2_P_2_O_7_)^2-^ anion displays three types of P‒O bond distances. The terminal P‒O_(terminal)_; the P‒OH; and the bridge P‒O_(bridge)_ bond distances range from 1.507(3) Å to 1.526(3) Å; from 1.567(3) to 1.567(3) Å and from 1.609(3) to 1.627(3) Å, respectively. The (H_2_P_2_O_7_)^2-^ anion shows bent eclipsed conformation, with a bridge P1‒O_4_‒P2 angle of 130.52(2)°. Both P‒O bond distances and bridge P‒O‒P angle values are comparable to similar diphosphates^33^.

**Figure 2:**
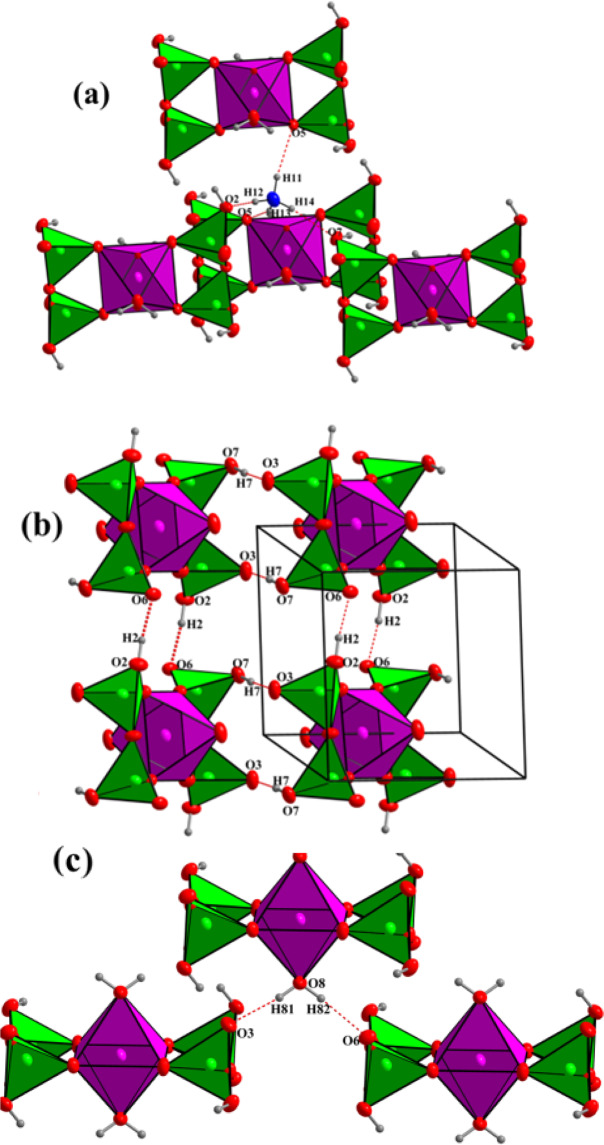
(**a**) View of the hydrogen bonds N-H^…^O, (**b**) P-O-H^…^O, and (**c**) Mg–O–H^…^O. The structural graphics were created with DIAMOND program.

**Figure 3:**
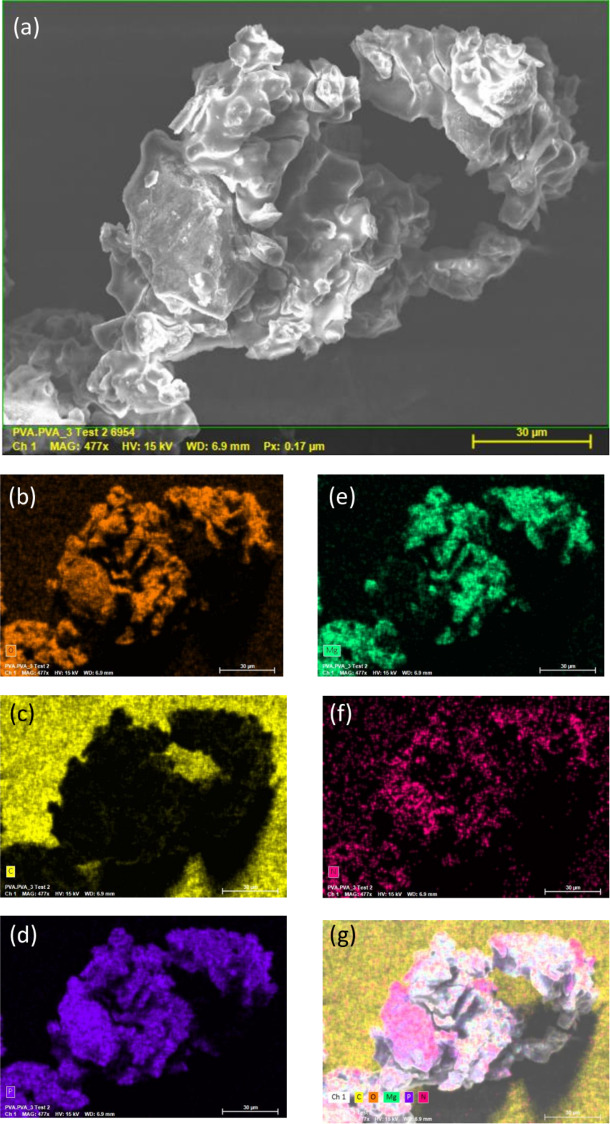
(**a**) Representative SEM and (**b–g**) associated EDS mapping of the elemental composition.

**Figure 4:**
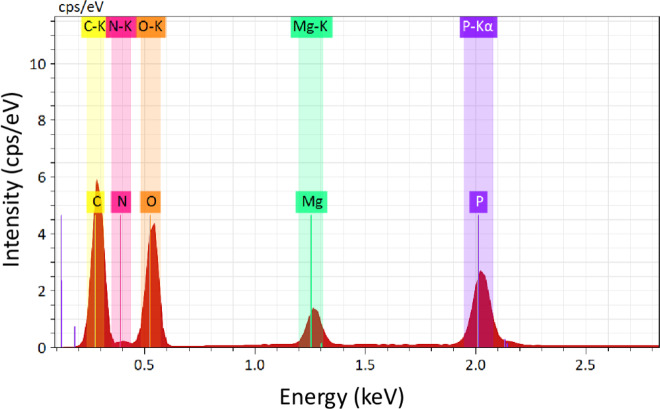
EDS spectrum (X-rays) of the elements on the compound (NH_4_)_2_Mg(H_2_P_2_O_7_)_2_•2H_2_O.

In the ammonium cation, the average N‒H distance is 0.897 Å, with the hydrogen pointing toward oxygen atoms of adjacent (H_2_P_2_O_7_)^2^ groups, forming a network of hydrogen bonds (see Table S6). Effectively, considering the H-bonds, [Mg(H_2_P_2_O_7_)_2_•2H_2_O]^2-^ anions turn in fact not isolated, they are interconnected through three types of moderate hydrogen bonds (i.e. P–O–H^…^O–P, Mg–O–H^…^O–P, and N–H^…^O–Mg) and form a stable 3D-framework. These hydrogen bonds include the H atoms from the ammonium cation (NH^4+^) (Fig. 2a), the hydroxyl groups (PO_3_OH) (Fig. 2b), and the water molecules (H_2_O) (Fig. 2c). Figure 3a shows a representative SEM micrograph of the (NH_4_)_2_Mg(H_2_P_2_O_7_)_2_•2H_2_O compound and (b-g) associated EDS elemental maps with elements O, C, P, Mg and N, respectively. Figure 4 shows the EDS spectrum of (NH_4_)_2_Mg(H_2_P_2_O_7_)_2_•2H_2_O. The atomic concentrations are shown Table S1.

### Vibrational spectroscopy

#### Factor group analysis

The Raman and infrared spectra of this compound have been collected and interpreted using factor group analysis. The crystallographic study shows that Mg cations are located on the 1*c* site, all the other ions are located on the (2*i*) sites. The irreducible representation of the compound in the C_i_ factor group (excluding 3 acoustic modes) leads to 57A_g_ (Ra) + 57A_u_ (IR) modes. The factor groups are centrosymmetric, the rule of mutual exclusion applies: the lines which are active in IR are not in Raman and vice versa. Note that Ag modes are Raman active and Au ones are infrared active.

The factor group analysis predicts the distribution of irreducible representation of the internal modes of (H_2_P_2_O_7_)^2-^, (NH_4_)^+^ ions and H_2_O molecules in the unit cell of the crystal (NH_4_)_2_Mg (H_2_P_2_O_7_)_2_•2H_2_O, to be respectively as follows:

$$\begin{array}{rcl}{\rm{G}}{({{\rm{H}}}_{2}{{\rm{P}}}_{2}{{\rm{O}}}_{7})}^{2-} & = & 27{\rm{Ag}}({\rm{Ra}})+27{{\rm{A}}}_{{\rm{u}}}({\rm{IR}})\\ {\rm{G}}{({{\rm{NH}}}_{4})}^{+} & = & 9{\rm{Ag}}({\rm{Ra}})+9{{\rm{A}}}_{{\rm{u}}}({\rm{IR}})\\ {\rm{G}}({{\rm{H}}}_{2}{\rm{O}}) & = & 3{\rm{Ag}}({\rm{Ra}})+3{{\rm{A}}}_{{\rm{u}}}({\rm{IR}})\end{array}$$

Tables S7‒S11 show the origin as well as a summary of the infrared and Raman activity of the internal and external modes of (H_2_P_2_O_7_)^2-^, H_2_O and (NH_4_)^+^, and Mg^2+^ in (NH_4_)_2_Mg(H_2_P_2_O_7_)_2_•2H_2_O.

### Interpretation of the Raman and infrared spectra

**Figure 5:**
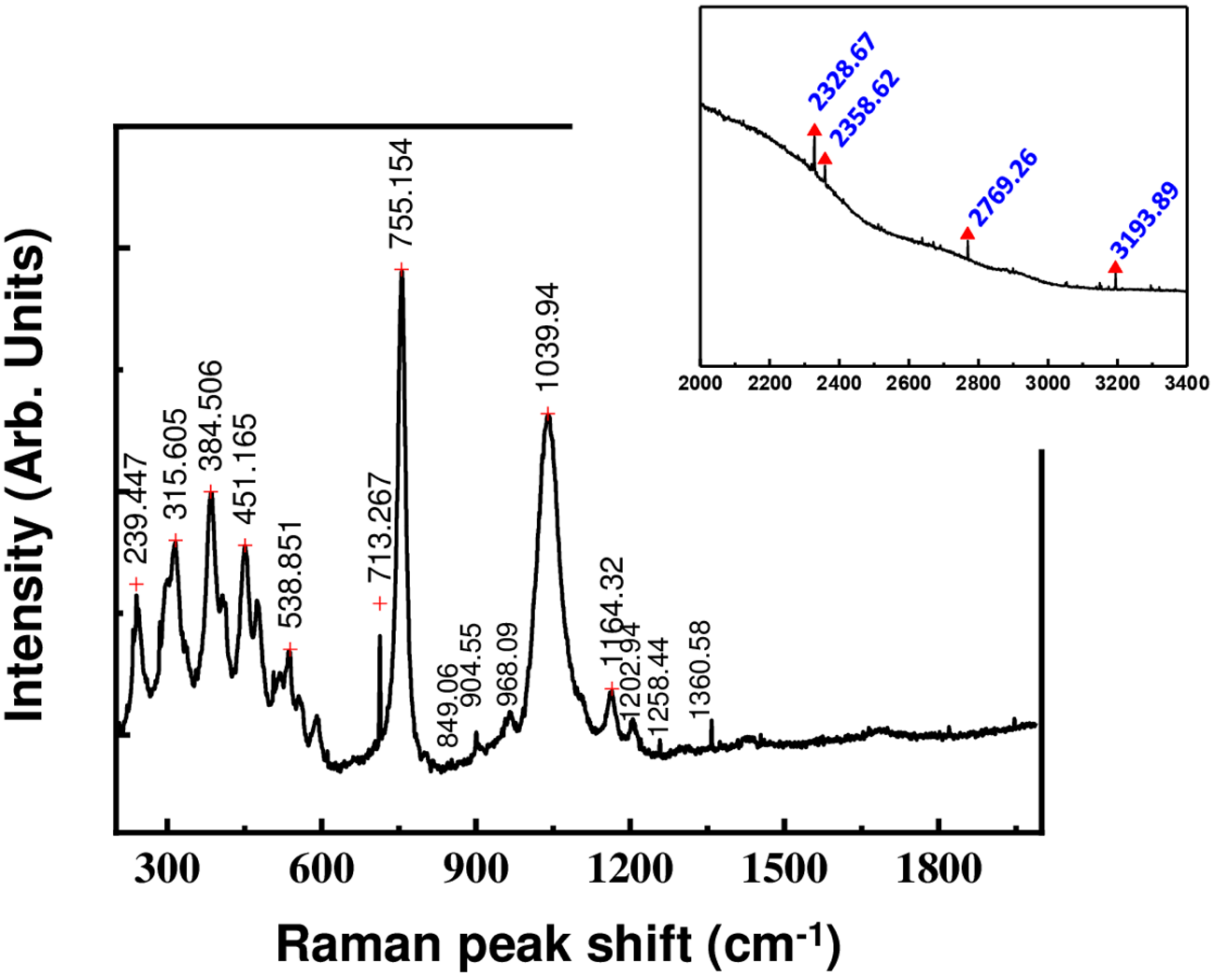
Raman spectrum of (NH_4_)_2_Mg(H_2_P_2_O_7_)_2_•2H_2_O compound showing the main bands locations.

**Figure 6:**
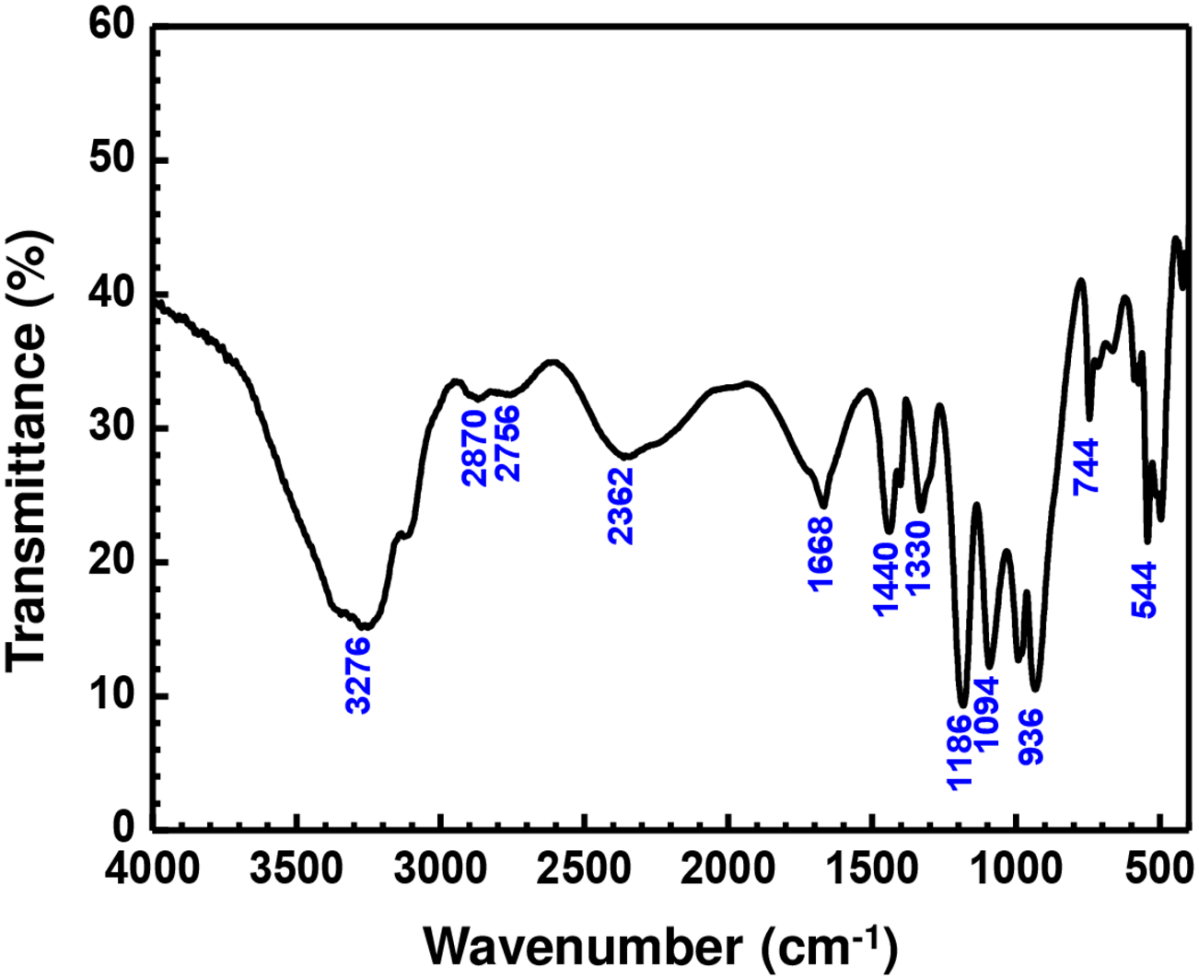
IR spectrum of (NH_4_)_2_Mg(H_2_P_2_O_7_)_2_•2H_2_O compound.

The interpretation of the Raman (Fig. 5) and infrared (Fig. 6) spectra can be made on the basis of characteristic vibrations of PO_2_ group, P‒OH bond, P‒O‒P bridge, H_2_O and (NH_4_)^+^ groups^10–12^. In both spectra, six strong bands are located at: 3744, 3346, 3276, 3110, 1670, 1446 and 1333 cm^−1^, while broad bands located in the region between 3744–3346 cm^−1^ correspond to the stretching vibration of the two water molecules (*v*(H_2_O)). The band falling in the region 3276–3110 cm^−1^ is associated to the *v*(NH_4_)^+^, while the band at 1670 cm^−1^ corresponds to the two water molecules and (NH_4_)^+^ ions [δ(H_2_O) and δ(NH_4_)^+^, respectively]. The bands at 1446–1333 cm^−1^ represent the bending bands of the ammonium ion. The frequencies of *v*(OH) are localized in the infrared spectrum in the range 3110–2362 cm^−1^, the observation of water molecules ρ(H_2_O) is only possible in the infrared spectrum^14^ at 662 cm^−1^. The band observed in the Raman spectrum at 1039.94 cm^−1^ (and 1094 cm^−1^ in the infrared spectrum) is attributed to the symmetric terminal P‒O stretching vibration of the PO_2_ group, and the band observed at around 1164 cm^−1^ in the Raman spectrum (and 1186 cm^−1^ in infrared spectrum) is due to the asymmetric terminal stretching vibration of the PO_2_ group. For the behavior of the P‒O‒P bridge vibrations, four components are observed in the Raman spectrum, γas(P‒O‒P) = 904 and 968 cm^−1^, γs(P‒O‒P) = 713 cm^−1^ and 755 cm^−1^, and three others in infrared spectrum at: γas(P‒O‒P) = 936 and 992 cm^−1^, γs(P‒O‒P) = 744 cm^−1^, which confirms the low symmetry of the cell (γ is the symmetric and/or asymmetric valence vibration modes)^10–17,34^. The band located at 849 cm^−1^ in Raman spectrum is due to the P‒OH mode^10–17^. The presence of γas(P‒O‒P) in the infrared spectrum and γas(P‒O‒P) in the Raman spectrum leads to a bent P‒O‒P bridge angle^34^. In the Raman spectrum, the modes lying between 239 and 384 cm^−1^ can be attributed to the external, torsional and P‒O‒P deformation modes. The δ(P‒O‒P) is observed at 315 cm^−1^, while the rocking vibration mode of the PO_2_ and the P-OH deformation mode are observed in the 300–594 cm^−1^ region^35^. A comparison of the Raman and infrared spectra shows that most of the bands do not coincide, which confirms a centrosymmetric structure of the (NH_4_)_2_Mg (H_2_P_2_O_7_)_2_•2H_2_O.

### CO_2_ and CH_4_ sorption

**Figure 7:**
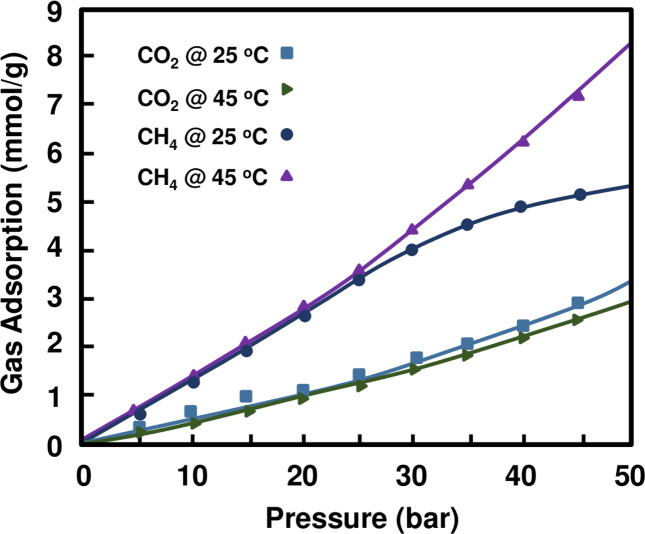
Plot of the CO_2_ and CH_4_ gas adsorption behaviour of (NH_4_)_2_Mg(H_2_P_2_O_7_)_2_•2H_2_O as a function of the pressure, with respect to temperatures of 25 and 45 °C, respectively.

The absorption and selectivity of (NH_4_)_2_Mg(H_2_P_2_O_7_)_2_•2H_2_O are evaluated by means of gravimetric analysis of the adsorption and desorption of CO_2_ and CH_4_ gases, which provides relevant information on the chemisorption versus physisorption behavior. Tests were performed at selected temperatures, namely 25 °C and 45 °C, while the pressure was varied from 0 to 50 bars. A stepwise absorption-desorption cycle was conducted by a programmed interval at each temperature. Buoyancy corrections on the sorption results -which operated through *in-situ* density measuring ability of magnetic suspension sorption apparatus (MSA) – were included. Additionally, external environmental conditions (humidity, ambient pressure and temperature) were considered automatically by MSA for zero-point corrections, to be measured with high accuracy to provide an authentic data set. Interestingly, no chemisorption/hysteresis was observed throughout our experiments as could be seen from the plots in Fig. 7 indicative of a preferred CO_2_ and CH_4_ physisorption of this material. The corresponding absorption-desorption values have been tabulated and made available in the supporting information (see Table S14 and Figures S1‒S4). The first noticeable remark is that the sorption increases with increasing pressure for both CO_2_ and CH_4_ gases. We recorded a maximum sorption of 8.29 mmol/g for CH_4_ at 45 °C and 50 bar while a lower value of 5.32 mmol/g at 25 °C was recorded at the same pressure. Lower sorption was recorder for CO_2_ with a maximum value not exceeding 2.96 mmol/g at 45 °C and 50 bar while a higher value of 3.38 mmol/g is measured at 25 °C. Atilhan and coworkers^36^ found values of the same order of magnitude by using magnetic suspension balance (MSB) system on Montmorillonite Nanoclays. The highest reported adsorption performance of CO_2_ versus CH_4_ at a temperature of 25 °C and a pressure of 50 bar was 3.47 mmol/g versus 3.23 mmol/g respectively. Interestingly, our material presents highly reproducible trends of high sorptivity of CH_4_ compared to CO_2_ at ambient temperature. Differences in CH_4_ vs. CO_2_ sorptivity become more pronounced with increasing temperature, a phenomenon deserving further experimental investigation to identify the exact mechanism governing it. For a comparison purpose, we have tested the CO_2_ and CH_4_ gas sorption at two different temperatures (25 & 45 °C) and pressure ranging from vacuum to 50 bar for the Rb_2_Co(H_2_P_2_O_7_)_2_.2H_2_O (unpublished results) where the preliminary results show that rate of CO_2_ and CH_4_ capturing is 3.10 mmol/g and 2.35 mmol/g at 25 °C and pressure of 50 bar, respectively.

### DFT calculations

**Figure 8:**
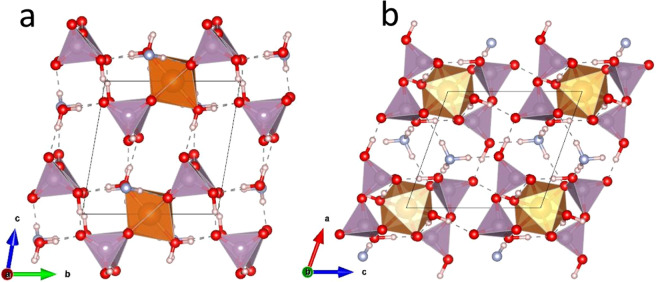
Ball and stick representation of the DFT relaxed cell of (NH_4_)_2_Mg(H_2_P_2_O_7_)_2_•2H_2_O viewed perpendicular to the (**a**) *a*-axis (**b**) *b*-axis. MgO_6_ octahedra and PO_4_ tetrahedra are shown in orange and purple respectively. Red: Oxygen, Light-blue: Nitrogen, Light pink: Hydrogen. Hydrogen bonds are shown as dashed lines. These structures were created by the open source software Vesta using 2D/3D visualisation crystal structures (http://jp-minerals.org/vesta/en/).

**Figure 9:**
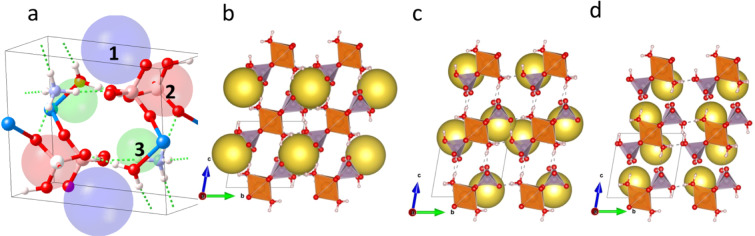
(**a**) Pores with radius> 1 Å identified in (NH_4_)_2_Mg(H_2_P_2_O_7_)_2_ unit cell. In panels (**b**), (**c**), (**d**), the large yellow spheres within a 2×2×2 supercell mark the location for pore 1, pore 2 and pore 3 respectively. These structures were created by the open source software Vesta using 2D/3D visualisation crystal structures (http://jp-minerals.org/vesta/en/).

The Density functional theory calculations show that the material is stable with lattice parameters and angles close to the resolved XRD structure as represented in Fig. 8. The DFT calculated lattice parameters are a = 7.057, b = 7.288, c = 7.732 Å and *α* = 80.56, *β* = 70.65, *γ* = 89.50°, and a volume of 369.79 Å^3^ in agreement with the experimentally reported structural data as displayed in Table S4. The pristine material is characterized by several pores with different coordination and radii as shown in Fig. 9a. A large pore (labeled 1) has a diameter of 3.4 Å forming a one-dimensional channel along the *a* direction. The spherical shape of pore 1 would be favorable to adsorb both CO_2_ and CH_4_ as shown in Fig. 9b. The subsequent pore 2 has a diameter of 2.9 Å forming a one-dimensional channel along the *b* direction as shown in Fig. 9c. Pore 3 shown in Fig. 9d is much smaller, with a diameter of 2.2 Å, and extends along the *c* direction. Another pore located in the center diameter 0.8 Å could be identified, however its elongated shape cannot accommodate large spherical molecules. Nevertheless, it could accommodate elongated molecules such as CO_2_, however the chemical environment and the coordination need to be favorable as well. Table S11 gives details about the identified pores with diameter> 2 Å. Combining this understanding of the morphology of the available pores to load molecules and the identified possible molecular diffusion pathways favor the hypothesis that pore 1 shall be loaded at first hand. We adopt an approach where we reassess the availability of open pores after each molecular loading. This is mainly motivated by our accurate understanding of the crystal structure featuring hydrogen bonds that are sensitive to the interaction molecules while -at the same time- giving an enhanced flexibility of bonding between the octahedral which is an advantage of this hybrid material. Thus, we cannot rule out that pore 1 could grow after the first loading and becomes favorable for accepting more molecules especially because of its 1D channel of diffusion parallel to the *a* direction.

**Figure 10:**
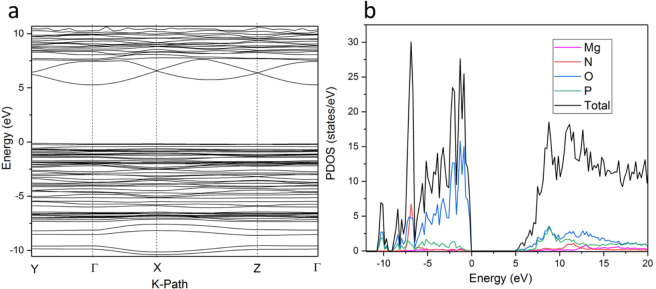
Calculated (**a**) Band structure and (**b**) Projected electronic density of states (PDOS) of (NH_4_)_2_Mg(H_2_P_2_O_7_)_2_•2H_2_O.

**Figure 11:**
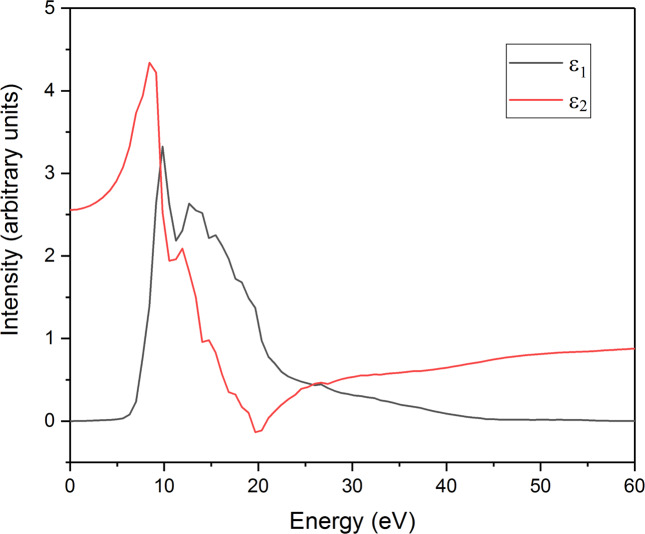
Calculated real (ε_1_) and imaginary part (ε_2_) of the complex dielectric function of (NH_4_)_2_Mg(H_2_P_2_O_7_)_2_•2H_2_O.

Figure 10 shows the calculated band structure of the pristine (NH_4_)_2_Mg(H_2_P_2_O_7_)_2_•2H_2_O. The system is an insulator with an indirect gap of 5.37 eV. The valence band maximum (VBM) is located near the R point displaying weakly dispersive flat bands. The conduction band minimum (CBM) is located at Γ point with well dispersed bands indicative of a strong overlap between molecular orbitals. Turning to the electronic density of states, Fig. 10a shows that Mg states contribute weakly to both VB and CB and far from the band edges. However, O *2p* orbitals dominated the VBM with some contribution form P 3*p* orbitals while the conduction band is hybridized between the O 2*p* and P *3p* orbitals with marginal contribution coming from other elements. The calculated real (ε1) and imaginary part (ε2) of the complex dielectric function are given in Fig. 11 also the Bader valence electron charges, charge transfer (relative to atoms), Bader volumes and the atomic partial charges in units of electron charges for DFT relaxed (NH_4_)_2_Mg(H_2_P_2_O_7_)_2_•2H_2_O are made available in Tables S15 and S16, respectively.

We proceeded by loading (NH_4_)_2_Mg(H_2_P_2_O_7_)_2_•2H_2_O with CO_2_ and CH_4_ molecules by targeting every time to occupy the largest available pores. In the case of the unloaded materials, each of the pore 1 sites (as shown in Fig. 9b) are occupied with a CO_2_ or a CH_4_ molecule.

**Figure 12:**
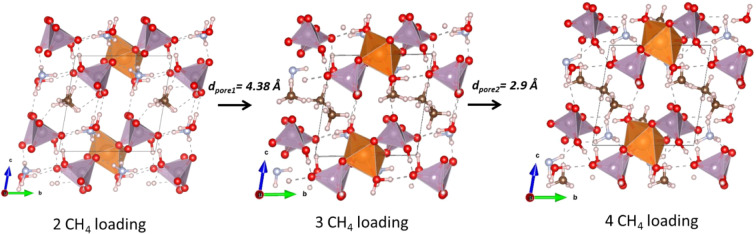
Schematic of sequences of CH_4_ loading in (NH_4_)_2_Mg(H_2_P_2_O_7_)_2_•2H_2_O. Hydrogen bonds are shown with dashed lines. *d*_*pore*_ indicates diameter of the next largest available pore ready to accept additional CH_4_ molecules. These structures were created by the open source software Vesta using 2D/3D visualisation crystal structures (http://jp-minerals.org/vesta/en/).

We loaded up to four CH_4_ molecules to occupy the large pore located between the P1 and P2 tetrahedra (pore 1), using the procedure described above. After each loading, the largest pore is identified then subsequently occupied as shown in the sequence illustrated in Fig. 12. It is noticeable from Fig. 12 that additional P‒O^…^H‒C hydrogen bonds form between the loaded CH_4_ molecule and the oxygen forming the PO_4_ tetrahedra. This newly formed hydrogen bonds in addition to the ones present in the pristine material (see Table S5) offers a significant flexibility of the material and might explain the increased capacity of loading CH_4_ by increasing temperature. We observe that loading the first two CH_4_ molecules allows to enlarge the diameter of pore 1 from *d*_*pore1*_ = 3.4 Å to *d*_*pore1*_ = 4.38 Å at the expense of slightly shrinking pore 2 and pore 3. Hence, it is more favorable to still load the third CH_4_ molecule within pore 1. Upon 3 CH_4_ molecules loading pore 1 reaches its maximum capacity, hence the next available pore to adsorb CH_4_ is pore 2 with a diameter *d*_*pore2*_ = 2.9 Å and diffusion pathway along the *b-*direction.

We proceed similarly to load the first two CO_2_ molecules at pore 1, it is noticeable that the CO_2_ molecule align to form Mg‒O‒H^…^O‒C hydrogen bonds with the [MgO_4_(H_2_O)_2_] octahedra avoiding the electrostatic repulsion originating from the neighboring PO_4_ tetrahedra. Both the dipole moment and the electrostatic directionality of the bonding suggest it pore 1 might not be able to accommodate further CO_2_ molecules. Indeed, after subjecting materials already loaded with two CO_2_ molecules to available pore searches, we identify the next loading position to be pore 2 with d_*pore2*_ = 3.0 Å. Additional loading with CO_2_ in the vicinity of PO_4_ tetrahedra seems to be energetically unfavorable as it of suffers from a strong electrostatic repulsion with the CO_2_ oxygen atoms. We loaded a third CO_2_ molecule which seems on average to adsorb less strongly to the pore 2.

Upon progressive addition of molecules to the largest available pore or so-called loading, it is noticeable that the material is more favorable to the adsorption of CH_4_ than CO_2_. Hence the sorption with CH_4_ is more favorable than that of CO_2_ in agreement with the experimental finding reported in the previous section. In light of our DFT calculations, the large sorption with CH_4_ can be attributed to formation of hydrogen bonds between the molecule and the PO_4_ tetrahedra contributing in bridging additional flexibility to the overall hydrogen bonding network. In contrast, CO_2_ loading is highly directional, as such CO_2_ molecule form hydrogen bonds with the [MgO_4_(H_2_O)_2_] octahedral while -at the same time- suffering from electrostatic repulsion with the PO_4_ tetrahedra which might be at the origin of the decreased sorption with CO_2_ observed experimentally. We speculate that this repulsion becomes more pronounced by increasing temperature from 25‒45 °C as observed experimentally. Although our calculations do not explicitly take into account the increase in temperature and pressure, assuming that under experimental loading conditions the properties of the pores and the atomic vibration are comparable regardless of the loaded molecule, the above finding remains valid.

### Antibacterial activity

**Figure 13:**
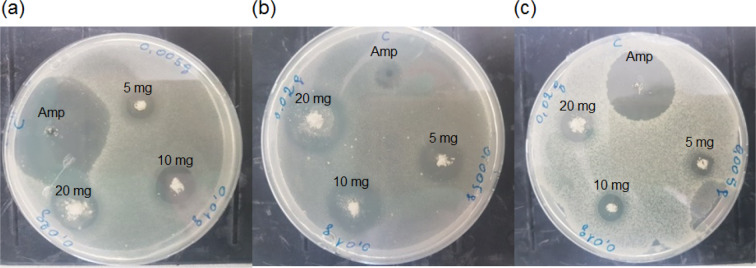
Inhibition zone of (NH_4_)_2_Mg(H_2_P_2_O_7_)_2_•2H_2_O compound against (**a**) *S*. *Aureus* (**b**) *E*. *Coli* and (**c**) *K*. *Pneumonia*. Amp: Ampicillin. Photo Credit: Dr. Souhir Sabri (QEERI).

The antibacterial assay revealed that (NH_4_)_2_Mg(H_2_P_2_O_7_)_2_•2H_2_O compound exhibits strong antibacterial activity against the tested Gram-positive and Gram-negative bacteria. According to Fig. 13 and Table S12, this material showed about 15 mm zone of inhibition against *E*. *Coli* at 5 mg, similarly, the same concentration showed 10 mm and 8 mm zone of inhibition against *S. Aureus* and *k. Pneumonia*, respectively. Higher concentration of (NH_4_)_2_Mg(H_2_P_2_O_7_)_2_•2H_2_O compound resulted an increase in antibacterial activity. At 20 mg, the compound showed approximately 23 mm, 17 mm and 14 mm against *E*. *Coli*, *S. Aureus* and *k. Pneumonia*, respectively. In sum, the antibacterial activity of the compound against *E*. *Coli* was stronger than *S*. *Aureus*, *K*. *Pneumonia* and the antibacterial property was increased with higher concentration of this material. This compound’s resistance to common bacterial growth makes it an attractive material for the adaptation into domestic air-conditioning greenhouse gas capturing and conversion units.

## Experimental and Methods

### Synthesis

(NH_4_)_2_Mg(H_2_P_2_O_7_)_2_•2H_2_O was successfully prepared using a wet method from stoichiometric mixtures of NH_4_Cl (Aldrich, ≥99%), Mg(NO_3_)_2_.6H_2_O (Aldrich, ≥99.99%), K_4_P_2_O_7_ (Aldrich, 99.99%) and HCl. First K_4_P_2_O_7_ was dissolved in 40 ml of water to form a clear solution then HCl was added (Solution A). The NH_4_Cl, and Mg(NO_3_)_2_.6H_2_O were dissolved together in 40 ml of H_2_O (Solution B). The solution (B) was then added dropwise to the solution (A) under continuous stirring. Afterwards it was left at room temperature, and crystals appeared within 3 days.

### X-ray crystallography

A crystal of (NH_4_)_2_Mg(H_2_P_2_O_7_)_2_•2H_2_O, rounded block 0.20 mm × 0.14 mm × 0.10 mm, was glued to a thin glass fiber and mounted on a OXFORD DIFFRACTION XCALIBUR four-circle X-ray diffractometer, equipped with graphite monochromatic MoKα radiation (λ = 0.7173 Å) and equipped with a SAPPHIR CCD two-dimensional detector. A total of 3014 reflections were collected (2θ_max_ = 26.37°) using the ω = 2θ scan mode. Of these 1905 are unique and 1587 were considered observed *I* > 2σ (*I)*. The intensity data were corrected for Lorentz and polarization effects. A numeric analytical absorption correction was carried out with the program CrysAlis RED^37^ Most of the positions of magnesium, phosphorus and oxygen atoms were located by direct methods, using the SHELXS-97 program^35^, and the remaining atoms were found from successive Fourier difference maps. Atomic positions were refined by fullmatrix least-squares method using SHELXL-97 program^38^. The non-hydrogen atoms were refined anisotropically. The H atoms were located geometrically, and attributed isotropic thermal factors equal to 1.2 those of the atoms on which they are linked. A final cycle refinement including atomic coordinates and anisotropic thermal parameters converged at R(F) = 0.0483 and wR(F^2^) = 0.1305 for the observed reflections. The unit cell parameters and data collection details are presented in Tables S2 and S3, respectively. The refined atomic positions and anisotropic ADPs are given in Tables S4 and S5, respectively. The structural graphics were created with DIAMOND program^39^. Further details on the structure refinements of (NH_4_)_2_Mg(H_2_P_2_O_7_)_2_•2H_2_O may be obtained from the Fachinformationszentrum Karlsruhe, D-76344 Eggenstein-Leopoldshafen (Germany), by quoting the Registry No. CSD-1988943.

### Raman spectroscopy

Details on the Raman spectroscopy can be found in our ref. ^12^. Briefly, the Raman spectroscopy was performed in air at room temperature, spectrum of (NH_4_)_2_Mg(H_2_P_2_O_7_)_2_•2H_2_O was collected in a back-scattering configuration via high throughput holographic imaging spectrograph equipped with volume transmission grating, holographic notch filter and thermoelectrically cooled CCD detector (Physics Spectra). The acquisition resolution was about 4 cm^−1^. Ti^3+^–sapphire NIR laser pumped by an argon ion laser was tuned at 785 nm. The laser was operated at a power not exceeding 40 mW to avoid any degradation of the samples, and the exposure time was about 60 s and 10 accumulations. A PERKIN-ELMER 1750 spectrophotometer was used for the infrared absorption analysis, in the 400‒4000 cm^−1^ range.

### Gas adsorption-desorption analysis

Rubotherm magnetic suspension sorption apparatus (MSA) allows the changes in force and mass which act on the sample that basically work on Archimedes’ buoyancy principle from high pressure to vacuum in order to complete gas absorption-desorption experiments. The data correlation, magnetic suspension force transmission error correction, calibration and working principle and methodology associated paraphernalia for broad range of adsorbents and absorbents were explained in detail in previous studies^40^. This apparatus contains *in-situ* density measurement capability with 4 kgm^−3^ uncertainty that makes possible for direct gravimetric measurements during the sorption experiments. Assembled pressure transducers (Paroscientific, USA) and temperature sensor (Minco PRT, USA) are capable to measure pressure up to 350 bars with an uncertainty of 0.01% of the full scale (u(p) ≈ 0.035 bar), and temperature with accuracy of ± 0.5 K (u(T) = 0.05 K), respectively. The automatic operated sorption apparatus starts measuring from vacuum to achieve high pressure through each pressure interval (as per given protocol) until the equilibrium is reached typically 45 to 60 min. MSA is operated to measure low pressure towards high pressure (adsorption) and then from high pressure to low pressure (desorption) including the end-of-sorption-cycle vacuum point measurement to be sure for chemisorption existence during the sorption cycle. The studied sample was well dried desiccated prior to use, and further vacuum was applied for at least 10 h before the sorption cycle initiated. The unchanged sample in the measurement bucket has given gas absorption-desorption data at isotherms of 25 and 45 °C.

### Microorganisms and inoculum preparation

The antimicrobial properties of the (NH_4_)_2_Mg(H_2_P_2_O_7_)_2_•2H_2_O compound were evaluated using *staphylococcus aur*eus as a model of Gram-positive bacteria and by using two other Gram-negative bacteria, namely *Escherichia Coli* and *Klebsiella Pneumonia*. For inoculum preparation, a single bacterial colony was picked from nutrient agar using disposable sterile loop, transferred into 10 mL of nutrient broth and placed overnight in incubator shaker at 37 °C with shaking speed of 100 rpm. The bacterial cell density was measured at an optical density (OD) of 600 nm using a spectro-photometer, each inoculum prepared would contains approximately 107 cfu/ml of bacteria. Bacterial sensitivity to (NH_4_)_2_Mg(H_2_P_2_O_7_)_2_•2H_2_O compound is performed by employing agar well diffusion method. Three-millimeter diameter holes were made in the agar plates using 50 ml disposable pipette and different concentration varying from 5 to 20 mg of this material was placed carefully in the holes, Ampicillin (10 µg/ml) was used as a standard antibiotic. The plates were overlaid with a mixture of each bacteria with 2 ml of molten 1.5% (w./vol.) noble agar (Sigma-Aldrich) at proximately 65 °C. Finally, the plates were incubated at 37 °C for 24 h and the average diameter of the inhibition zone surrounding the holes was examined.

### Density Functional Theory (DFT) calculations

Density functional theory (DFT) calculations were employed to study the properties of (NH_4_)_2_Mg(H_2_P_2_O_7_)_2_ porous material. Spin polarized DFT GGA calculation was used the Perdew-Burke-Ernzerhof functional (PBE) as implemented in the Vienna ab-initio simulation package^41–47^ (VASP) with the projector augmented wave (PAW) pseudopotentials. Due to the presence of hydrogen bonds, Van Der Waals (vdW) interactions where taken into account via Tkatchenko-Scheffler (TS) scheme^48,49^. Full structural optimization of the unit cell was performed until convergence criteria for optimizations reached 10^–3^ eV and 10^–4^ eV for the ionic relaxation loop and self-consistent electronic iteration, respectively. A kinetic cutoff energy of 520 eV for the plane waves has been employed and the Brillouin zone was sampled by Monkhorst-Pack grid centered at the Gamma point with a k-point mesh of 8×8×8.

## Conclusion

diammonium magnesium dihydrogendiphosphate (V) dehydrate (NH_4_)_2_Mg(H_2_P_2_O_7_)_2_•2H_2_O was successfully synthesized by a wet chemical process, and characterized in detail by X-ray diffraction, FTIR and micro Raman spectroscopy. This compound was found to crystallize in the triclinic system, space group *P*−1 (No.2) with *a* = 7.007(9) Å, *b* = 7.339(1) Å, *c* = 7.796(1) Å, *α* = 81.240(1) °, *β* = 71.080(1)°, *γ* = 88.150(1)°, *V* = 374.78(9) Å^3^, Z = 1. It crystal structure was mainly built of isolated [Mg(H_2_P_2_O_7_)_2_•2H_2_O]^2-^ anions separated by NH_4_^+^ cations. The Mg^2+^ ion is positioned on the inversion center and the MgO_6_ octahedra share four vertices with the dihydrogen diphosphate anion (H_2_P_2_O_7_)^2-^ that showed bent eclipsed conformation.

The compound was then tested for CO_2_ and CH_4_ storage applications and showed higher sorptivity with CH_4_ than with CO_2_ at 25 °C and 45 °C under pressures up to 50 bars. DFT calculations were also employed to compute the principal characteristics of identified pores in this compound, including the projected electronic density of states, band structures, the complex dielectric function, as function of the progressive CO_2_ vs. CH_4_ loadings. An antibacterial assay was also performed and has shown a strong antibacterial activity against the tested Gram-positive and Gram-negative bacteria, with a large zone of inhibition against the tested *E*. *Coli*, *S. Aureus* and *K. Pneumonia*, demonstrating thereby a real potential for preventing the proliferation of infectious diseases. The multidisciplinary analysis presented in this work demonstrated clearly the potential of this material for the selective capture of CH_4_/CO_2_ gases opening the way for its applications in conjunction with carbon capture from air conditioning systems in houses/buildings and for energy applications at large (e.g. liquid hydrocarbon fuels production).

## Supplementary information

Supplementary information.

## References

[1] Andreeva OI (2001). Ya. Interaction of HIV-1 Reverse Transcriptase and T7 RNA Polymerase with Phosphonate Analogs of NTP and Inorganic Pyrophosphate. Molecular Biology.

[2] Mathew M, Schroeder LW, Brown WE (1993). Crystal structure of dicalcium potassium trihydrogen bis(pyrophosphate) trihydrate. Journal of Crystallographic and Spectroscopic Research.

[3] Byrappa K (1994). Crystal growth, morphology, structure, and properties of HNaMP_2_O_7_ crystals where M = Co and Ni. J. Mater. Res..

[4] Corbridge DEC (1957). Crystallographic data on some hypophosphates and pyrophosphates. Acta Crystallographica.

[5] Collin RL, Willis M (1971). The crystal structure of disodium dihydrogen hypophosphate hexahydrate (Na_2_H_2_P_2_O_6_.6H_2_O) and disodium dihydrogen pyrophosphate hexahydrate (Na_2_H_2_P_2_O_7_.6H_2_O). Acta Crystallographica B.

[6] Dumas Y, Galigne JL, Falgueirettes J (1973). Structure cristalline de l’hydrogéno pyrophosphate tripotassique trihydraté, K_3_HP_2_O_7_.3H_2_O. Acta Crystallographica B.

[7] Durif A. (1995). Mixed-Anion Phosphates. Crystal Chemistry of Condensed Phosphates.

[8] Harcharras M (2003). Synthesis, X-ray crystal structure and vibrational spectroscopy of the acidic pyrophosphate KMg_0.5_H_2_P_2_O_7_.H_2_O. Journal of Solid State Chemistry.

[9] Assaaoudi H (2002). KZn(HP_2_O_7_)·2H_2_O and KMn(HP_2_O_7_)·2H_2_O: acid pyrophosphate metallates(II) with layer structures. Acta Crystallographica C.

[10] Essehli R (2005). Synthesis, crystal structure and vibrational spectra of a new diammonium zinc(II) dihydrogendiphosphate dihydrate, (NH_4_)_2_Zn(H_2_P_2_O_7_)_2_·2H_2_O. Acta Crystallographica.

[11] Essehli R (2005). Elbali. (NH_4_)_2_[Co(H_2_P_2_O_7_)_2_(H_2_O)_2_]. Acta Crystallographica E6.

[12] Essehli, R., et al. (NH_4_)_2_[Ni(H_2_P_2_O_7_)_2_(H_2_O)_2_]. Acta Crystallographica E61, i64–i66 (2005).

[13] Alaoui. AT (2004). Dipotassium nickel(II) bis(dihydrogendiphosphate) dihydrate, K_2_Ni(H_2_P_2_O_7_)_2_·2H_2_O. Acta Crystallographica.

[14] Tahiri AA (2003). Dipotassium zinc bis(dihydrogendiphosphate) dihydrate, K_2_Zn(H_2_P_2_O_7_)_2_·2H_2_O. Acta Crystallographica E.

[15] Capitelli F (2004). Two new ammonium diphosphates: crystal structure of Mn_0.5_NH_4_H_2_P_2_O_7_ center dot H_2_O and MnNaNH_4_P_2_O_7_ center dot 3H_2_O. Zeitschrift Fur Kristallographie.

[16] Tahiri AA (2002). Cobalt potassium dihydrogendiphosphate dihydrate, CoK_2_(H_2_P_2_O_7_)_2_·2H_2_O. Acta Crystallographica E.

[17] El Bali B (2008). New thallium diphosphates Tl_2_Me(H_2_P_2_O_7_)_2_· 2H_2_O, Me = Mg, Mn, Co, Ni and Zn. Synthesis, single crystal X-ray structures and powder X-ray structure of the Mg phase. Zeitschrift Fur Kristallographie.

[18] Ben Saad A (2016). A New Photoluminescent Co(II)-Diphosphate Cluster Templated by Fampridine Cation: Synthesis and Biophysicochemical Evaluation. Journal of Cluster Science.

[19] Lashof DA, Ahuja DR (1990). Relative Contributions of Greenhouse Gas Emissions to Global Warming. Nature.

[20] Kitagawa S (2015). Porous Materials and the Age of Gas. Angewandte Chemie.

[21] Bao Z (2011). Adsorption Equilibria of CO_2_, CH_4_, N_2_, O_2_, and Ar on High Silica Zeolites. J. Chem. Eng. Data.

[22] Amhamed A, Atilhan M, Berdiyorov G (2019). Permeabilities of CO_2_, H_2_S and CH_4_ through Choline-Based Ionic Liquids: Atomistic-Scale Simulations. J. Mol..

[23] Abotaleb A, El-Naas MH, Amhamed A (2017). Enhancing gas loading and reducing energy consumption in acid gas removal systems: A simulation study based on real NGL plant data. J. Nat. Gas Sci. Eng..

[24] Altamash T (2019). Combined Experimental and Theoretical Study on High Pressure Methane Solubility in Natural Deep Eutectic Solvents. Ind. & Eng. Chem. Res..

[25] Liu Y, Wilcox J (2012). Effects of Surface Heterogeneity on the Adsorption of CO_2_ in Microporous Carbons. Environ. Sci. Technol..

[26] Le Page M (2012). Determination of the Solid–Liquid Phase Diagram of the Binary System Acrylic Acid + Propionic Acid. Journal of Chemical & Engineering Data.

[27] Coudert FO-X (2009). Prediction of Breathing and Gate-Opening Transitions Upon Binary Mixture Adsorption in Metal−Organic Frameworks. J. Am. Chem. Soc..

[28] Sumida K (2012). Carbon dioxide capture in metal-organic frameworks. Chem. Rev..

[29] Altamash T (2017). Carbon dioxide solubility in phosphonium-, ammonium-, sulfonyl-, and pyrrolidinium-based ionic liquids and their mixtures at moderate pressures up to 10 bar. J. Chem. Eng. Data.

[30] Altamash T (2017). Gas solubility and rheological behavior of natural deep eutectic solvents (NADES) via combined experimental and molecular simulation techniques. Chemistry Select.

[31] Altamash T (2017). Rheological, Thermodynamic, and Gas Solubility Properties of Phenylacetic Acid‐Based Deep Eutectic Solvents. Chem. Eng. Technol..

[32] Altamash T (2018). Gas solubility and rheological behavior study of betaine and alanine based natural deep eutectic solvents (NADES). Journal of Molecular Liquids.

[33] Lager GA, Gibbs GV (1973). Effect of variations in OPO and POP angles on PO bond overlap populations for some selected ortho-and pyrophosphate. American Mineralogist.

[34] Khaoulaf R (2017). Vibrational Spectra of Dizincate Sodium Triphosphte Nonahydrate Zn2NaP3O10.9H2O. Journal of Materials and Environmental Sciences.

[35] Sheldrick, G. M. SHELXS-97, program for the solution of crystal structures, University of Göttingen, Germany (1990).

[36] Atilhan M (2016). High-Pressure Methane, Carbon Dioxide, and Nitrogen Adsorption on Amine-impregnated Porous Montmorillonite Nanoclays. J. Chem. Eng. Data.

[37] Clark R. C. & Reid J. S. CrysAlis RED, Oxford Diffraction Ltd., Program for analytical numeric absorption correction, Version 170.17, (2003).

[38] Sheldrick, G. M. SHELXL-97, program for crystal structure determination, University of Göttingen, Germany (1997).

[39] Brandenburg, K. DIAMOND, Crystal Impact GbR, Bonn, Germany, Version 2.1 e, (2001).

[40] Coudert FO-X (2009). Prediction of Breathing and Gate-Opening Transitions Upon Binary Mixture Adsorption in Metal-Organic Frameworks. J. Am. Chem. Soc..

[41] Kresse GJ, Furthmüller J (1996). Efficient iterative schemes for ab initio total-energy calculations using a plane-wave basis set. Phys. Rev. B.

[42] Kresse G, Furthmuller J (1996). Efficiency of ab initio total energy calculations for metals and semiconductors using a plane-wave basis set. Comput. Mater. Sci..

[43] Kresse G, Hafner J (1993). Ab initio molecular dynamics for liquid metals. Phys. Rev. B.

[44] Kresse G, Hafner J (1994). Ab initio molecular-dynamics simulation of the liquid-metal–amorphous-semiconductor transition in germanium. Phys. Rev. B.

[45] Perdew JP, Burke K, Ernzerhof M (1996). Generalized Gradient Approximation Made Simple. Phys. Rev. Lett..

[46] Blöchl PE (1994). Projector augmented-wave method. Phys. Rev. B.

[47] Kresse G, Joubert D (1999). From ultrasoft pseudopotentials to the projector augmented-wave method. Phys. Rev. B.

[48] Tkatchenko A, Scheffler M (2009). Accurate Molecular Van Der Waals Interactions from Ground-State Electron Density and Free-Atom Reference Data. Phys. Rev. Lett..

[49] Yang J (2012). Adsorption of CO_2_, CH_4_, and N_2_ on gas diameter grade ion-exchange small pore zeolites. J. Chem. Eng. Data.

