# Supplementary material for: Single crystal structure, vibrational spectroscopy, gas sorption and antimicrobial properties of a new inorganic acidic diphosphates material (NH4)2Mg(H2P2O7)2•2H2O
Source: Sci Rep. 2020 Jun 1;10:8909. doi: 10.1038/s41598-020-65718-2 (PMC7264148; doi:10.1038/s41598-020-65718-2)
Supplement: Supplementary file 1 — Supplementary information. [file 41598_2020_65718_MOESM1_ESM.docx]

# Single crystal structure, vibrational spectroscopy, gas sorption and antimicrobial properties of a new inorganic acidic diphosphates material (NH_4_)_2_Mg(H_2_P_2_O_7_)_2_•2H_2_O

Rachid Essehli^*,1^, Souhir Sabri^2^, Fedwa El-Mellouhi^2^, Brahim Aïssa*^,2^, Hamdi Ben Yahia^2^, Tausif Altamash^2^, Majeda Khraisheh^3^ , Brahim El Bali^4^ & Abdulkarem Amhamed^*,2^

*^1^Energy and Transportation Science Division, Oak Ridge National Laboratory, Oak Ridge, TN, USA.*

*^2^Qatar Environment & Energy Research institute (QEERI), Hamad Bin Khalifa University (HBKU), Qatar Foundation, P.O. Box 34110 Doha, Qatar.*

*^3^ Chemical Engineering Department, Qatar University, P.O. Box 2713 Doha, Qatar.*

*^4^Independent scientist.*

**Corresponding authors*: B. Aïssa (QEERI), E-mail: [baissa@hbku.edu.qa](mailto:baissa@hbku.edu.qa); R. Essehli (ORNL), E-mail: [essehlir@ornl.gov](mailto:essehlir@ornl.gov); A. Amhamad (QEERI), E-mail: [aamhamad@hbku.edu.qa](mailto:aamhamad@hbku.edu.qa)

**Supporting Information**

Tables:

**Table S1.** EDS Elemental analysis of (NH_4_)_2_Mg(H_2_P_2_O_7_)_2_•2H_2_O.

| **Element** | ***Mass %*** | ***Atm. %*** |
| --- | --- | --- |
| Carbon | 47.68 | 55.41 |
| Nitrogen | 3.99 | 3.98 |
| Oxygen | 43.46 | 37.92 |
| Phosphorus | 0.89 | 0.40 |
| Magnesium | 3.97 | 2.28 |
| Sum | 100 | 100 |

**Table S2.** A summary of Unit cell parameters of (*MTPy*•*n*H_2_O) family of compounds reported in the literature.

| **Compound** | **S.G.** | ***a* (Å)** | ***b* (Å)** | ***c* (Å)** | α **(°)** | β **(°)** | γ **(°)** | **Ref** |
| --- | --- | --- | --- | --- | --- | --- | --- | --- |
| (NH_4_)_2_Zn(H_2_P_2_O_7_)_2_•2H_2_O | *P\-1* | 7.0074 | 7.339 | 7.796 | 81.24 | 71.08 | 88.15 | [10] |
| (NH_4_)_2_Ni(H_2_P_2_O_7_)_2_•2H_2_O | *P\-1* | 7.0344 | 7.321 | 7.792 | 81.53 | 70.91 | 88.210 | [11] |
| (NH_4_)_2_Co(H_2_P_2_O_7_)_2_•2H_2_O | *P\-1* | 7.0670 | 7.368 | 7.852 | 81.23 | 70.68 | 88.540 | [12] |
| K_2_Ni(H_2_P_2_O_7_)_2_•2H_2_O | *P\-1* | 6.8554 | 7.3124 | 7.5610 | 81.012 | 72.301 | 83.691 | [13] |
| K_2_Zn(H_2_P_2_O_7_)_2_•2H_2_O | *P\-1* | 6.827 | 7.333 | 7.570 | 80.753 | 72.547 | 83.442 | [14] |
| K_2_Mg(H_2_P_2_O_7_)_2_•2H_2_O | *P\-1* | 6.8565 | 7.3621 | 7.6202 | 81.044 | 72.248 | 83.314 | [8] |
| (NH_4_)_2_Mn(H_2_P_2_O_7_)_2_•2H_2_O | *P\-1* | 7.0029 | 7.4401 | 7.8771 | 80.444 | 71.359 | 87.408 | [15] |
| K_2_Co(H_2_P_2_O_7_)_2_•2H_2_O | *Pnma* | 9.7044 | 11.0023 | 13.3937 | 90 | 90 | 90 | [16] |
| K_2_Mn(H_2_P_2_O_7_)_2_•2H_2_O | *Pnma* | 9.7613 | 11.162 | 13.394 | 90 | 90 | 90 | [14] |
| Na_2_Mg(H_2_P_2_O_7_)_2_•4H_2_O | *P*2_1_/*m* | 8.0445 | 11.5244 | 9.0825 | 90 | 113.14 | 90 | [14] |
| Tl_2_Mn(H_2_P_2_O_7_)_2_•2H_2_O | P\-1 | 6.9577 | 7.4745 | 7.8248 | 80.72 | 71.912 | 85.66 | [17] |
| Tl_2_Co(H_2_P_2_O_7_)_2_•2H_2_O | *P\-1* | 6.9767 | 7.3634 | 7.7690 | 81.421 | 71.114 | 86.424 | [17] |
| Tl_2_Ni(H_2_P_2_O_7_)_2_•2H_2_O | *P\-1* | 6.9658 | 7.3079 | 7.7016 | 81.801 | 71.185 | 86.528 | [17] |
| Tl_2_Zn(H_2_P_2_O_7_)_2_•2H_2_O | *P\-1* | 6.9628 | 7.3625 | 7.7401 | 81.590 | 71.441 | 86.323 | [17] |

**Table S3.** Crystallographic and structure refinements data for (NH_4_)_2_Mg(H_2_P_2_O_7_)_2_•2H_2_O.

| **Crystal data** | |
| --- | --- |
| Chemical formula | (NH_4_)_2_Mg(H_2_P_2_O_7_)_2_•2H_2_O |
| *M*_r_ | 448.34 |
| Crystal system, space group | Triclinic, *P-*1 |
| Temperature (K) | 302 |
| *a*, *b*, *c* (Å) | 7.076 (2), 7.431 (1), 7.894 (2) |
| α, β, γ (°) | 81.42 (2), 70.90 (3), 87.78 (2) |
| *V* (Å^3^) | 387.82 (16) |
| *Z* | 1 |
| Radiation type | Mo *K*α |
| µ (mm^−1^) | 0.61 |
| Crystal size (mm) | 0.20 × 0.14 × 0.10 |
| Data collection |  |
| Diffractometer | Oxford Diffraction Xcalibur (TM) Single Crystal X-ray Diffractometer with Sapphire CCD Detector. |
| Absorption correction | Analytical |
| *T*_min_, *T*_max_ | 0.910, 0.950 |
| No. of measured, independent and |  |
| observed [*I* > 2σ(*I*)] reflections | 2560, 1542, 1337 |
| *R*_int_ | 0.045 |
| (sin θ/λ)_max_ (Å^−1^) | 0.625 |
| Refinement |  |
| *R*[*F*^2^ > 2σ(*F*^2^)], *wR*(*F*^2^), *S* | 0.048, 0.134, 1.16 |
| No. of reflections | 1542 |
| No. of parameters | 130 |
| No. of restraints | 14 |
| H-atom treatment | H-atom parameters constrained |
| Δρ_max_, Δρ_min_ (e Å^−3^) | 0.66, −0.44 |

**Table S4**. Atomic positions and isotopic displacement parameters (Å^2^) for (NH_4_)_2_Mg(H_2_P_2_O_7_)_2_•2H_2_O.

| **Atom** | **Wyck.** | ***x*** | ***y*** | ***z*** | ***U_iso_* (Å^2^)** |
| --- | --- | --- | --- | --- | --- |
| Mg1 | 1*c* | 0 | 1/2 | 0 | 0.0197(4) |
| P1 | 2*i* | 0.25866(14) | 0.20695(13) | -0.25534(13) | 0.0182(3) |
| P2 | 2*i* | -0.15554(14) | 0.25811(13) | -0.25469(13) | 0.0186(3) |
| O1 | 2*i* | 0.2422(4) | 0.3511(4) | -0.1357(4) | 0.0273(6) |
| O2 | 2*i* | 0.4419(4) | 0.2371(4) | -0.4342(4) | 0.0312(7) |
| H2 | 2*i* | 0.552(5) | 0.265(7) | -0.410(7) | 0.0370 |
| O3 | 2*i* | 0.2508(5) | 0.0117(4) | -0.1609(4) | 0.0345(7) |
| O4 | 2*i* | 0.0805(4) | 0.2274(4) | -0.3449(4) | 0.0260(6) |
| O5 | 2*i* | -0.1870(4) | 0.3825(4) | -0.1108(4) | 0.0226(6) |
| O6 | 2*i* | -0.2288(4) | 0.3303(4) | -0.4117(4) | 0.0281(7) |
| O7 | 2*i* | -0.2517(5) | 0.0664(4) | -0.1639(4) | 0.0305(7) |
| H7 | 2*i* | -0.244(8) | 0.039(7) | -0.051(3) | 0.0370 |
| O8 | 2*i* | 0.0640(5) | 0.7111(4) | -0.2220(4) | 0.0332(7) |
| H81 | 2*i* | 0.121(7) | 0.806(4) | -0.214(6) | 0.0400 |
| H82 | 2*i* | 0.107(7) | 0.695(6) | -0.332(2) | 0.0400 |
| N1 | 2*i* | -0.4157(6) | 0.7118(5) | -0.2040(5) | 0.0318(8) |
| H11 | 2*i* | -0.536(3) | 0.680(5) | -0.122(4) | 0.0380 |
| H12 | 2*i* | -0.418(5) | 0.701(5) | -0.315(2) | 0.0380 |
| H13 | 2*i* | -0.322(4) | 0.640(5) | -0.178(5) | 0.0380 |
| H14 | 2*i* | -0.386(6) | 0.827(3) | -0.201(5) | 0.0380 |

**Table S5**. Interatomic distances (Å), Angles (^o^) and bond valences (B.V.) of (NH_4_)_2_Mg(H_2_P_2_O_7_)_2_•2H_2_O: XRD data side to side with those obtained using DFT calculations. Average distances and angles are given in brackets.

|  | **XRD** | | | **DFT** | | |
| --- | --- | --- | --- | --- | --- | --- |
|  | ***Distances(Å)*** | ***Angles(°)*** | | ***Distances (Å)*** | ***Angles(°)*** | |
| Mg1-O1 | 2.077(3) | O1-P1-O3 | 114.99 (18) | 2.053 | O1-P1-O3 | 115.04 |
| Mg1-O1 | 2.077(3) | O1-P1-O2 | 113.22 (17) | 2.053 | O1-P1-O2 | 113.34 |
| Mg1-O5 | 2.086(3) | O3-P1-O2 | 110.89 (19) | 2.110 | O3-P1-O2 | 110.74 |
| Mg1-O5 | 2.086(3) | O1-P1-O4 | 110.65 (16) | 2.110 | O1-P1-O4 | 111.22 |
| Mg1-O8 | 2.110(3) | O3-P1-O4 | 107.14 (17) | 2.060 | O3-P1-O4 | 106.75 |
| Mg1-O8 | 2.110(3) | O2-P1-O4 | 98.54 (16) | 2.060 | O2-P1-O4 | 98.30 |
|  | <2.091> |  | <109.24> | <2.074> |  | <109.23> |
|  |  |  |  |  |  |  |
| P1-O1 | 1.507(3) | O6-P2-O7 | 109.94 (17) | 1.503 | O6-P2-O7 | 110.05 |
| P1-O3 | 1.523(3) | O5-P2-O7 | 109.84 (17) | 1.529 | O5-P2-O7 | 109.40 |
| P1-O2 | 1.567(3) | O6-P2-O4 | 105.17 (16) | 1.568 | O6-P2-O4 | 104.07 |
| P1-O4 | 1.627(3) | O5-P2-O4 | 108.86 (15) | 1.625 | O5-P2-O4 | 109.16 |
|  | <1.556> | O7-P2-O4 | 106.84 (17) | <1.556> | O7-P2-O4 | 107.47 |
|  |  |  |  |  |  |  |
| P2-O6 | 1.627(3) | P2-O4-P1 | 130.51 (19) | 1.5128 | P2-O4-P1 | 129.79 |
| P2-O5 | 1.514(3) |  |  | 1.524 |  |  |
| P2-O7 | 1.526(3) |  |  | 1.569 |  |  |
| P2-O4 | 1.567(3) |  |  | 1.615 |  |  |
|  | <1.559> |  |  | <1.555> |  |  |
|  |  |  |  |  |  |  |
| N1-H11 | 0.897(9) |  |  | 1.034 |  |  |
| N1-H12 | 0.897(9) |  |  | 1.044 |  |  |
| N1-H13 | 0.895(10) |  |  | 1.038 |  |  |
| N1-H14 | 0.897(10) |  |  | 1.038 |  |  |
|  | <0.897> |  |  | <1.038> |  |  |

^a^ bond valence sum, B.V. = e^(^*^r^*^0–^*^r^*^)/b^ with the following parameters: *b* = 0.37, *r*_0_ (Mg^II^–O) = 1.693, and *r*_0_ (P^V^–O) = 1.617[37,38].

**Table S6**. Hydrogen-bond geometry (Å, º).

| ***D*—H···*A*** | ***D*—H** | **H···*A*** | ***D*···*A*** | ***D*—H···*A*** |
| --- | --- | --- | --- | --- |
| O2—H2···O6^i^ | 0.90 (1) | 1.64 (2) | 2.525 (4) | 168 (5) |
| O7—H7···O3^ii^ | 0.90 (1) | 1.65 (1) | 2.545 (4) | 175 (5) |
| O8—H81···O3^iii^ | 0.85 (1) | 1.97 (1) | 2.817 (4) | 172 (4) |
| O8—H82···O6^iv^ | 0.85 (1) | 1.96 (1) | 2.801 (4) | 174 (4) |
| N1—H11···O5^v^ | 0.90 (1) | 2.22 (1) | 3.103 (5) | 171 (3) |
| N1—H12···O2^iv^ | 0.90 (1) | 2.02 (2) | 2.890 (5) | 162 (3) |
| N1—H13···O5 | 0.90 (1) | 2.17 (2) | 3.014 (5) | 156 (4) |
| N1—H14···O7^iii^ | 0.90 (1) | 2.15 (1) | 3.019 (5) | 164 (4) |

Symmetry codes: (i) *x*+1, *y*, *z*; (ii) −*x*, −*y*, −*z*; (iii) *x*, *y*+1, *z*; (iv) −*x*, −*y*+1, −*z*−1; (v) −*x*−1, −*y*+1, −*z*.

**Table S7**. Selected bands from Raman and IR spectra (NH_4_)_2_Mg (H_2_P_2_O_7_)_2_•2H_2_O.

| **Wavelength Raman (cm^-1^)** | **Wavelength IR (cm^-1^)** | **Assignment** |
| --- | --- | --- |
|  | 3744–3346 | ν (H_2_O) |
|  | 3276–3110 | ν (NH_4_)+ |
|  | 2362 | ν (OH) |
| 1621.72 | 1670 | δ (H_2_O) |
| 1433.45 | 1333-1446 | δ (NH_4_)^+^ |
| 1257.67, 1205.08 and 1164.32 | 1186 | νs (PO_2_) |
| 1039.94 | 1094 | νas (PO_2_) |
| 986.09 and 904.5 | 992 and 936 | νs(P-O-P) stretches |
| 849.06 | 842 | ν (P-OH) |
| 755.154 and 713.267 | 744 | νas(P-O-P) stretch |
| 594.022-239.447 | 496-662 | region of P-O bending modes, NH_4_ tensional oscillation |

ν: stretching; δ: deformation; as: asymmetric; s: symmetric

**Table S8**. Correlation scheme for the internal modes of (H_2_P_2_O_7_)^2-^ in (NH_4_)_2_Mg (H_2_P_2_O_7_)_2_•2H_2_O (Ra: Raman; IR: Infrared)

| **Free ion group (C2V)** | **Site group (C1)** | **Factor group (Ci)** |
| --- | --- | --- |
| 9A_1_ (Ra, IR)  5A_2_ (Ra, IR)  5B_1_ (Ra, IR)  8B_2_ (Ra, IR) | 27A (Ra, IR) | 27Ag (Ra)  27Au (IR) |
| Raman 27  Infrared 22  Coincidence 22 | 27  27  27 | 27  27  0 |

**Table S9**. Correlation scheme for the internal modes of (NH_4_)^+^ in (NH_4_)_2_Mg (H_2_P_2_O_7_)_2_•2H_2_O (Ra: Raman; IR: Infrared).

| **Free ion group ((Td)** | **Site group (C1)** | **Factor group (Ci)** |
| --- | --- | --- |
| 1A_1_(Ra)  1E(Ra)  2T_2_( Ra, IR) | 9A(Ra, IR) | 9A_g_(Ra)  9A_u_( IR) |
| Raman 4  Infrared 2  Coincidence 2 | 9  9  9 | 9  9  0 |

**Table S10**. Correlation scheme for the internal modes of (H_2_O) in (NH_4_)_2_Mg (H_2_P_2_O_7_)_2_•2H_2_O (Ra: Raman; IR: Infrared).

| **Free ion Group (C2V)** | **Site group (C1)** | **Factor group (Ci)** |
| --- | --- | --- |
| 2A1 ( Ra, IR)  1B2 (Ra, IR) | 3A(Ra, IR) | 3A_g_(Ra)  3A_u_( IR) |
| Raman 3  Infrared 3  Coincidence 3 | 3  3  3 | 3  3  0 |

**Table S11.** Correlation scheme for the external modes of (H_2_P_2_O_7_)^2-^, Mg^2+^, NH_4_ and H_2_O in (NH_4_)_2_Mg(H_2_P_2_O_7_)_2_•2H_2_O

| External modes  R T | G_S_= C_1_ | G_F_ = C_i_ | External modes  R T | |
| --- | --- | --- | --- | --- |
| 3 0  H_2_P_2_O_7_^2-^  0 3 | A (IR, Ra) | A_g_ (Ra)  A_u_ (IR) | | 3 3  3 3 |
| 3 0  H_2_O  0 3 | A(Ra, IR) | A_g_ (Ra)  A_u_ (IR) | | 3 3  3 3 |
| 3 0  NH_4_^+^  0 3 |  | A_g_ (Ra)  A_u_ (IR) | | 3 3  3 3 |
|  | A(IR, Ra) |  |  |  |
| 0 -  Mg^2+^  - 3 | G_S_= C_i_ | G_F_= Ci  A_g_ (Ra)  A_u_ (IR) | | - 0  - 3 |
|  | A_g_(Ra)  A_u_(IR) |  |  |  |

R= Rotation, T= Translation, G_s_= Site group, G_F_ = Factor group. IR: infrared; Ra: Raman

**Table S12**. Principal characteristics of identified pores in (NH_4_)_2_Mg(H_2_P_2_O_7_)_2_ with pore diameter > 2 Å.

| **Pore Id** | **Diameter (Å)** | **Site** | **Coordination** | **Position X** | **Position Y** | **Position Z** |
| --- | --- | --- | --- | --- | --- | --- |
| 1 | 3.46 | 2i | 5 | 0.2592 | 0.9978 | 0.4894 |
| 2 | 2.75 | 2i | 4 | 0.4723 | 0.1982 | 0.8951 |
| 3 | 2.22 | 2i | 3 | 0.6921 | 0.3806 | 0.2464 |

**Table S13.** Antibacterial Activity data of (NH_4_)_2_Mg (H_2_P_2_O_7_), 2H_2_O compound.

| **Bacteria** | **Zone of inhibition (mm)** |
| --- | --- |
| Cronobacter Sokazakii | 7 |
| Aeromonas Veronii | 1 |
| Enterobacter Sacchari | 6 |
| Kluyvera Crycrescens | 6 |
| Pseudomonas Aeruginosa | 2 |
| Bacillus Subtilis | 8 |

**Table S14**. Atomic coordinates and isotropic displacement parameters for (NH_4_)_2_Mg(H_2_P_2_O_7_)_2_•2H_2_O.

| Atoms | ***U_11_*** | ***U_22_*** | ***U_33_*** | ***U_23_*** | ***U_13_*** | ***U_12_*** |
| --- | --- | --- | --- | --- | --- | --- |
| Mg1 | 0.0240 (9) | 0.0166 (8) | 0.0195 (9) | -0.0055 (7) | -0.0071 (7) | 0.0004 (7) |
| P1 | 0.0165 (5) | 0.0205 (5) | 0.0183 (5) | -0.0051 (4) | -0.0055(4) | 0.0014 (3) |
| P2 | 0.0171 (5) | 0.0205 (5) | 0.0201 (5) | -0.0053 (4) | -0.0075 (4) | 0.0004 (3) |
| O1 | 0.0270 (15) | 0.0308 (15) | 0.0292 (15) | -0.0145 (12) | -0.0120 (12) | 0.0042 (12) |
| O2 | 0.0220 (15) | 0.0475 (19 | 0.0248 (15) | -0.0100 (13) | -0.0062 (12) | -0.0019 (13 |
| O3 | 0.050(2) | 0.0208 (15 | 0.0352 (17) | -0.0001 (12) | -0.0191 (15) | -0.0030 (13 |
| O4 | 0.0178 (14) | 0.0351 (16) | 0.0289 (15) | -0.0115 (12) | -0.0102 (12) | 0.0038 (11) |
| O5 | 0.0219 (14) | 0.0214 (13 | 0.0262 (14) | -0.0087 (11) | -0.0082 (12) | 0.0038 (10) |
| O6 | 0.0238 (15) | 0.0371 (17 | 0.0247 (15) | 0.0001 (12) | -0.0115 (12) | -0.0016 (12) |
| O7 | 0.0380 (17 | 0.0241 (15 | 0.0314 (16) | -0.0023 (12) | -0.0137 (14) | -0.0085 (12 |
| O8 | 0.052(2) | 0.0228 (15 | 0.0221 (15) | -0.0022 (12) | -0.0074 (14) | -0.0058 (13 |
| N1 | 0.038(2) | 0.032(2) | 0.0262 (18) | -0.0055 (15) | -0.0106 (16) | 0.0073 (16) |

**Table S15.** CO_2_ / CH_4_ absorption values in mmol/g of (NH_4_)_2_Mg(H_2_P_2_O_7_)_2_•2H_2_O (A = Adsorption; D. =Desorption) at different pressures and temperatures.

| ***T = 25 °C*** | | | | ***T = 45 °C*** | | | |
| --- | --- | --- | --- | --- | --- | --- | --- |
| **P/ bar** | **mmol/g CO_2_ (A)** | **P/ bar** | **mmol/g CO_2_ (D)** | **P/ bar** | **mmol/g CO_2_ (A)** | **P/ bar** | **mmol/g CO_2_ (D)** |
| 0.0497 | 0.0105824 | 49.9174 | 3.3858253 | 0.0501 | 0.0117788 | 49.9122 | 2.9656898 |
| 0.9304 | 0.0506296 | 40.0668 | 2.4752252 | 0.9371 | 0.052297 | 40.0609 | 2.2415936 |
| 4.9413 | 0.2373803 | 30.0668 | 1.7004692 | 4.9400 | 0.238433 | 30.0575 | 1.5755319 |
| 9.9339 | 0.4824172 | 20.075 | 1.0668625 | 9.9333 | 0.4794027 | 20.0724 | 1.0051301 |
| 14.9315 | 0.7451811 | 10.0582 | 0.5098114 | 14.9382 | 0.7313245 | 10.0538 | 0.4862885 |
| 19.9303 | 1.0269178 | 5.051 | 0.2544379 | 19.9319 | 0.9952035 | 5.0515 | 0.244201 |
| 24.9246 | 1.3298601 | 1.4427 | 0.0769376 | 24.9317 | 1.2728152 | 1.4089 | 0.0728552 |
| 29.9199 | 1.6572386 | 0.0488 | 0.0117509 | 29.9277 | 1.5654585 | 1.05 | 0.0574346 |
| 34.9474 | 2.0416946 | 0.0499 | 0.0118365 | 34.9647 | 1.9001902 | 0.0489 | 0.0116729 |
| 39.9309 | 2.4363686 |  |  | 39.9499 | 2.2307372 |  |  |
| 44.9518 | 2.8812001 |  |  | 44.9321 | 2.5848622 |  |  |
| 49.9174 | 3.3858253 |  |  | 49.9122 | 2.9656898 |  |  |
| ***T = 25 °C*** | | | | ***T = 45 °C*** | | | |
| **P/ bar** | **mmol/g CH_4_ (A)** | **P/ bar** | **mmol/g CH_4_ (D)** | **P/ bar** | **mmol/g CH_4_ (A)** | **P/ bar** | **mmol/g CH_4_ (D)** |
| 0.0492 | 0.0323576 | 49.9386 | 5.3233644 | 0.0507 | 0.0282285 | 49.9305 | 8.2909785 |
| 0.913 | 0.1451439 | 40.0552 | 4.8800083 | 0.9258 | 0.139951 | 40.0664 | 6.2513754 |
| 4.9319 | 0.6811779 | 30.0915 | 3.9618137 | 4.9416 | 0.6586366 | 30.0951 | 4.3889751 |
| 9.9433 | 1.3645524 | 20.0888 | 2.7481304 | 9.9354 | 1.3305001 | 20.0617 | 2.7954838 |
| 14.939 | 2.0486826 | 10.0615 | 1.3763859 | 14.923 | 2.0320555 | 10.0576 | 1.3506407 |
| 19.9244 | 2.7299176 | 5.0506 | 0.5827393 | 19.9074 | 2.7682513 | 5.0523 | 0.6772933 |
| 24.9212 | 3.388737 | 1.358 | 0.1695259 | 24.9094 | 3.5423318 | 1.4489 | 0.2009675 |
| 29.9163 | 3.9796158 | 0.0489 | 0.0296933 | 29.9322 | 4.3599566 | 0.0491 | 0.1583104 |
| 34.9271 | 4.5051514 | 0.0499 | 0.0296878 | 34.9124 | 5.2931143 | 0.0503 | 0.0308572 |
| 39.9206 | 4.8689071 |  |  | 39.9403 | 6.2220485 |  |  |
| 44.9501 | 5.1330408 |  |  | 44.9153 | 7.2125144 |  |  |
| 49.9386 | 5.3233644 |  |  | 49.9305 | 8.2909785 |  |  |

**Table S16.** Calculated Bader valence electron charges, charge transfer (relative to atoms) and Bader volumes for DFT calculated (NH_4_)_2_Mg(H_2_P_2_O_7_)_2_•2H_2_O.

| **Atom** | **Valence charge** | **Charge transfer** | **Volume (Å ^3^)** | **Distance Å** |
| --- | --- | --- | --- | --- |
| Mg2 | 0.3049 | 1.6951 | 4.8321 | 0.7910 |
| P1 | 1.3368 | 3.6632 | 3.5448 | 0.1749 |
| P2 | 1.3219 | 3.6781 | 3.4994 | 0.1342 |
| O1 | 7.5062 | -1.5062 | 18.1480 | 0.7892 |
| O2 | 7.4356 | -1.4356 | 18.5505 | 0.7544 |
| H2 | 0.3286 | 0.6714 | 1.2544 | 0.1080 |
| O3 | 7.4619 | -1.4619 | 17.2794 | 0.8292 |
| O4 | 7.5262 | -1.5262 | 17.1301 | 0.8853 |
| O5 | 7.5055 | -1.5055 | 15.8625 | 0.8093 |
| O6 | 7.4615 | -1.4615 | 18.5728 | 0.7960 |
| O7 | 7.4481 | -1.4481 | 17.7328 | 0.7997 |
| H7 | 0.3340 | 0.6660 | 1.2152 | 0.1176 |
| O8 | 7.3240 | -1.3240 | 18.9304 | 0.7114 |
| H81 | 0.3494 | 0.6506 | 1.6483 | 0.0909 |
| H82 | 0.3494 | 0.6506 | 1.7046 | 0.1034 |
| N1 | 6.2466 | -1.2466 | 17.1005 | 0.7086 |
| H11 | 0.4923 | 0.5077 | 2.6946 | 0.1658 |
| H12 | 0.4674 | 0.5326 | 2.4536 | 0.1564 |
| H13 | 0.4816 | 0.5184 | 2.4911 | 0.1624 |
| H14 | 0.4705 | 0.5295 | 2.6656 | 0.1325 |
| Vacuum |  | 0.0000 | -0.0000 | 0.0031 |
| Total |  | 144.0000 | 0.0000 | 369.79 |

**Table S17.** Atomic partial charges in units of electron charges for DFT relaxed (NH_4_)_2_Mg(H_2_P_2_O_7_)_2_•2H_2_O.

| **Atom** | **s** | **p** | **d** | **Total** |
| --- | --- | --- | --- | --- |
| Mg2 | 0.262 | 0.404 | 0.269 | 0.935 |
| P1 | 0.850 | 1.416 | 0.655 | 2.921 |
| P2 | 0.847 | 1.415 | 0.657 | 2.919 |
| O1 | 1.255 | 2.925 | 0.011 | 4.191 |
| O2 | 1.244 | 2.965 | 0.011 | 4.221 |
| H2 | 0.122 | 0.006 | 0.000 | 0.128 |
| O3 | 1.253 | 2.926 | 0.010 | 4.190 |
| O4 | 1.244 | 2.965 | 0.011 | 4.220 |
| O5 | 1.253 | 2.924 | 0.010 | 4.187 |
| O6 | 1.253 | 2.931 | 0.011 | 4.195 |
| O7 | 1.246 | 2.952 | 0.011 | 4.209 |
| H7 | 0.120 | 0.006 | 0.000 | 0.126 |
| O8 | 1.233 | 2.989 | 0.011 | 4.233 |
| H81 | 0.145 | 0.006 | 0.000 | 0.151 |
| H82 | 0.146 | 0.006 | 0.000 | 0.152 |
| N1 | 0.967 | 2.310 | 0.019 | 3.297 |
| H11 | 0.154 | 0.004 | 0.000 | 0.158 |
| H12 | 0.149 | 0.004 | 0.000 | 0.153 |
| H13 | 0.151 | 0.004 | 0.000 | 0.156 |
| H14 | 0.151 | 0.004 | 0.000 | 0.156 |

**Figures:**

**
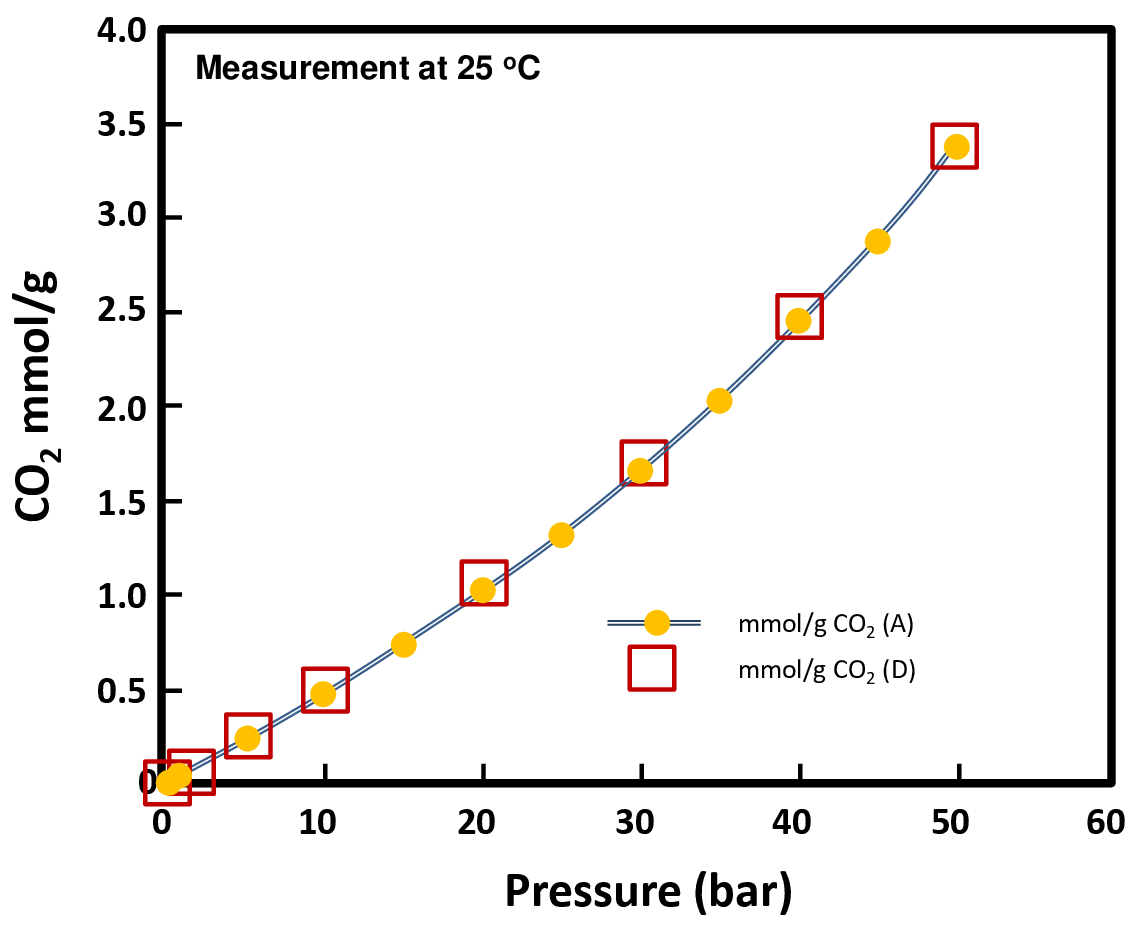
**

**Figure S1.** CO_2_ solubility (absorption (A), desorption (D)) in (NH_4_)_2_Mg(H_2_P_2_O_7_)_2_•2H_2_O at 25 °C and different pressures.


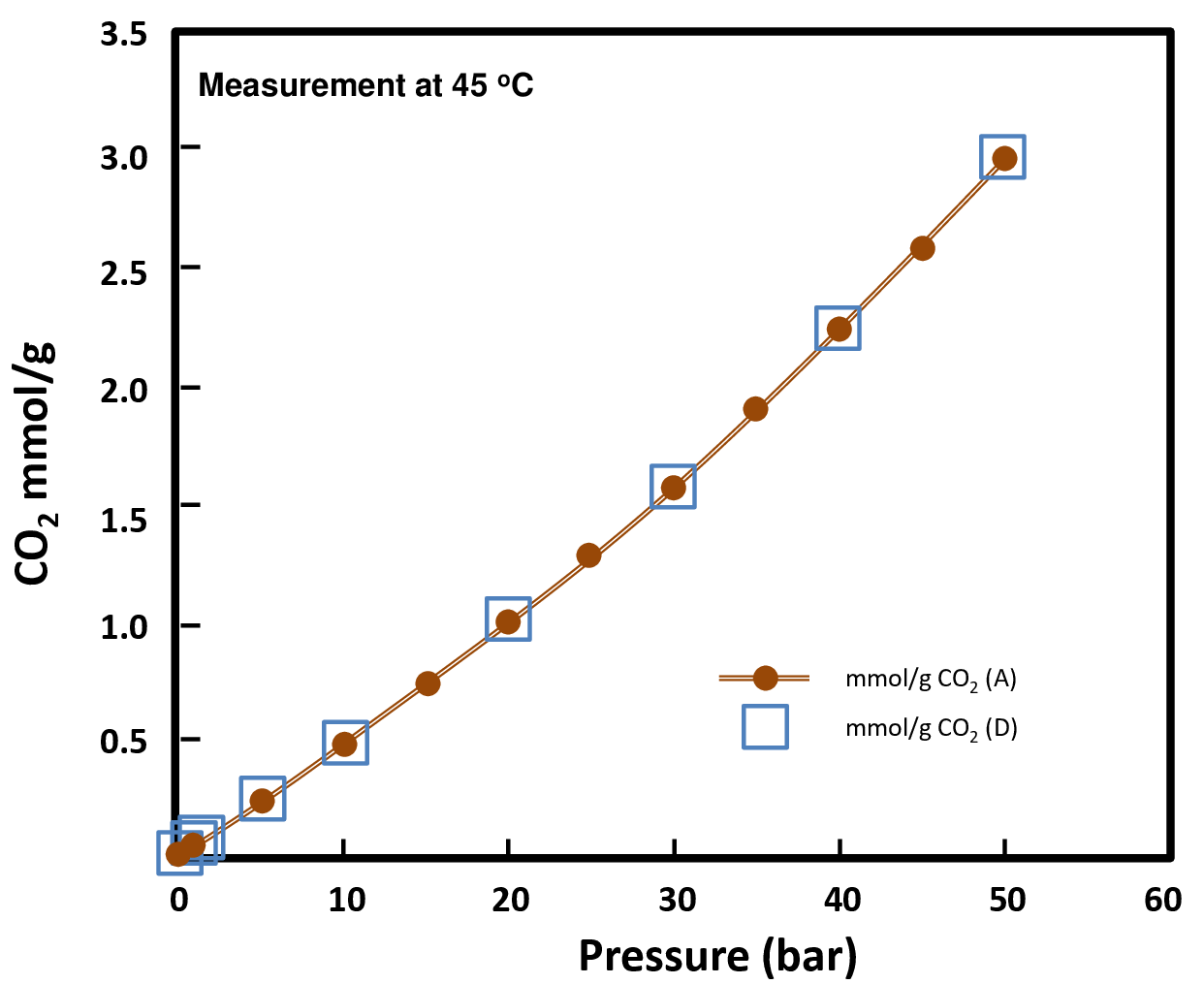


**Figure S2.** CO_2_ solubility in (NH_4_)_2_Mg(H_2_P_2_O_7_)_2_•2H_2_O at 45 °C and different pressures.

**
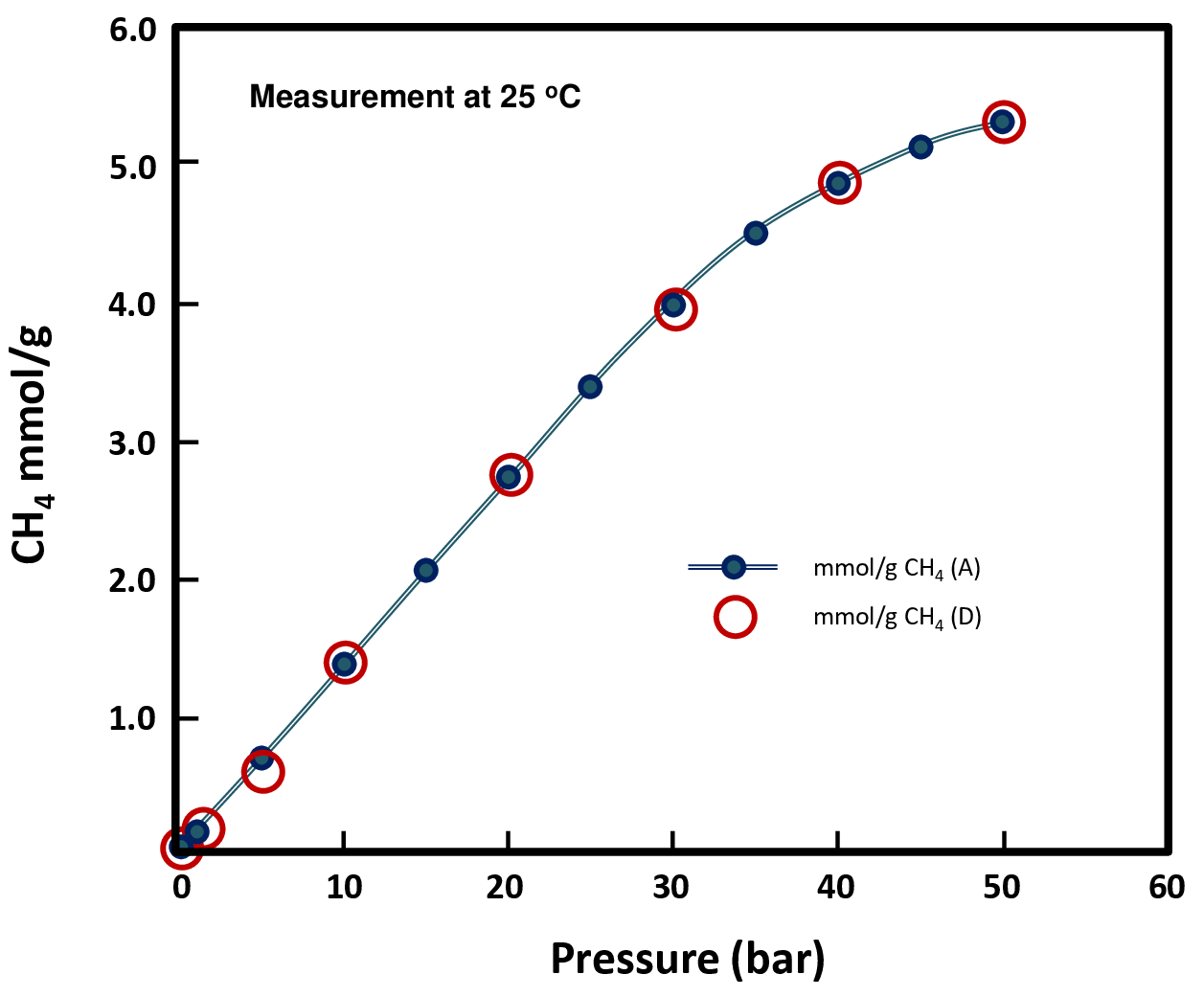
**

**Figure S3.** CH_4_ solubility in (NH_4_)_2_Mg(H_2_P_2_O_7_)_2_•2H_2_O at 25 °C and different pressures.

**
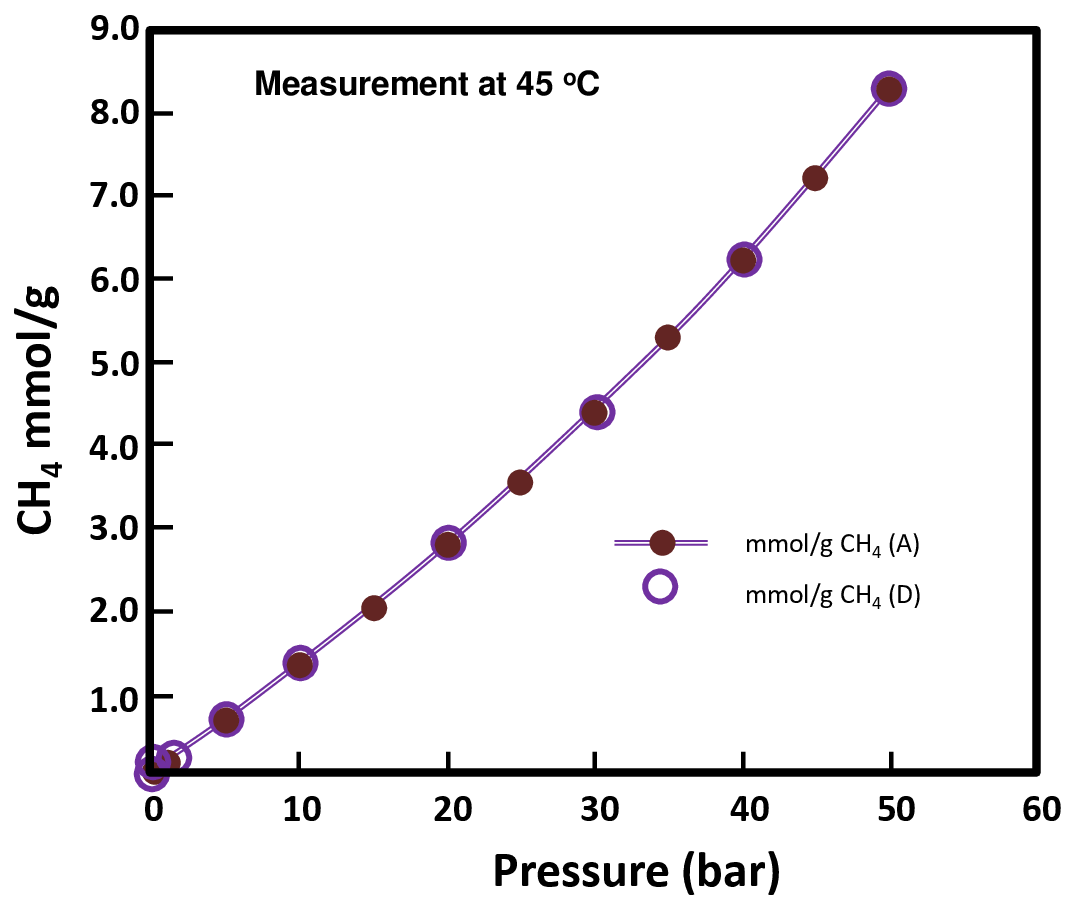
**

**Figure S4.** CH_4_ solubility in (NH_4_)_2_Mg(H_2_P_2_O_7_)_2_•2H_2_O at 45 °C and different pressures.
